# Role of plate convergence rate in shaping earthquake recurrence in subduction zones

**DOI:** 10.1038/s41598-025-04766-y

**Published:** 2025-07-01

**Authors:** Sayak Ray, Bhaskar Kundu, Batakrushna Senapati, Abhijit Ghosh, Arun K. Singh

**Affiliations:** 1 Department of Earth and Atmospheric Sciences, NIT Rourkela, Rourkela, 769008 India; 2 Department of Earth Science, National Central University, No. 300, Jhongda Rd, Chungli, Taoyuan, 320 Taiwan; 3 Department of Earth and Planetary Sciences, University of California Riverside, Riverside, USA; 4 Department of Mechanical Engineering, VNIT, Nagpur, 440010 Maharashtra India

**Keywords:** Slow slip events, Megathrust earthquakes, Rate and state friction, Cascadia subduction zone, Nankai subduction zone, Stress-meters, Geology, Geophysics, Tectonics

## Abstract

Understanding the complex interplay of subduction zone processes is key to unravelling the timing and distribution of great earthquake cycles within the framework of the plate tectonics paradigm. Megathrust earthquakes, though extensively investigated and their quasi-repetitive nature well recognised, remain challenging to assess globally due to their long recurrence intervals and limited historical data. Slow earthquakes in the brittle-to-ductile transition zone interact dynamically with megathrust events, but their potential to trigger or delay large earthquakes remains unclear. The periodic nature of slow earthquakes (typically recurring over timescales of months to years) has enabled detailed seismic and geodetic catalogs, offering new insights into subduction zone dynamics. Here, we adopt a tripartite approach, integrating natural observations, numerical simulations, and laboratory experiments to investigate relationship between megathrust and slow earthquakes in subduction zones. Analysis of tremor catalogs of Cascadia and Nankai subduction zones, reveal a systematic logarithmic inverse relationship between recurrence intervals (T_r_) and plate convergence rates (V_L_), with downdip tremor patches exhibiting shorter recurrence times than updip segments. Quasidynamic rate-and-state friction (RSF) simulations, calibrated against geodetic displacements, demonstrate that this periodicity arises from frictional healing modulated by V_L_ (T_r_ ∞ -V_L_*)*. Laboratory stick-slip experiments validate this scaling, showing force drop and recurrence times decrease logarithmically with increasing loading velocity, consistent with natural and numerical observations. Crucially, the logarithmic dependency persists across tectonic (months–years), numerical (stick-slip cycles), and laboratory (seconds–minutes) scales, resolving ambiguities in scaling fault mechanics from lab to tectonic regimes. We propose that SSEs in the brittle-ductile transition zone episodically transfer stress to adjacent locked megathrust segments, acting as real-time ‘stress-meters’ for seismogenic zone dynamics. This framework bridges geophysical monitoring, Rate and State Friction theory, and experimental fault mechanics, offering a predictive tool to infer stress accumulation on megathrusts. By linking slow earthquake periodicity to plate kinematics, our findings advance a unified paradigm for earthquake cycle dynamics, directly informing probabilistic hazard models and mitigation strategies in subduction zones. The integration of multi-scale constraints underscores the potential of tremor networks to monitor stress evolution, enhancing our capacity to identify regions at risk of large seismic events.

**Supplementary Information:**

The online version contains supplementary material available at 10.1038/s41598-025-04766-y.

## Introduction

Megathrust faults along convergent plate boundaries cause devastating damage due to strong ground shaking caused by great earthquakes and possible tsunamis. Understanding their seismic potential, rupture behaviour, stress accumulation and eventually, stress release process during the interseismic, coseismic and postseismic periods on the shallow offshore segment of the subduction megathrust faults, along with the retrieval of a precursory signal prior to the megathrust earthquake, are the critical motivation for the seismic/tsunami induced hazards and disaster mitigation. Although diverse scientific efforts have been directed toward earthquake prediction^1–5^unfortunately, not a single approach/method has been established for the short-term (or long-term) prediction of an impending giant megathrust.

**Fig. 1:**
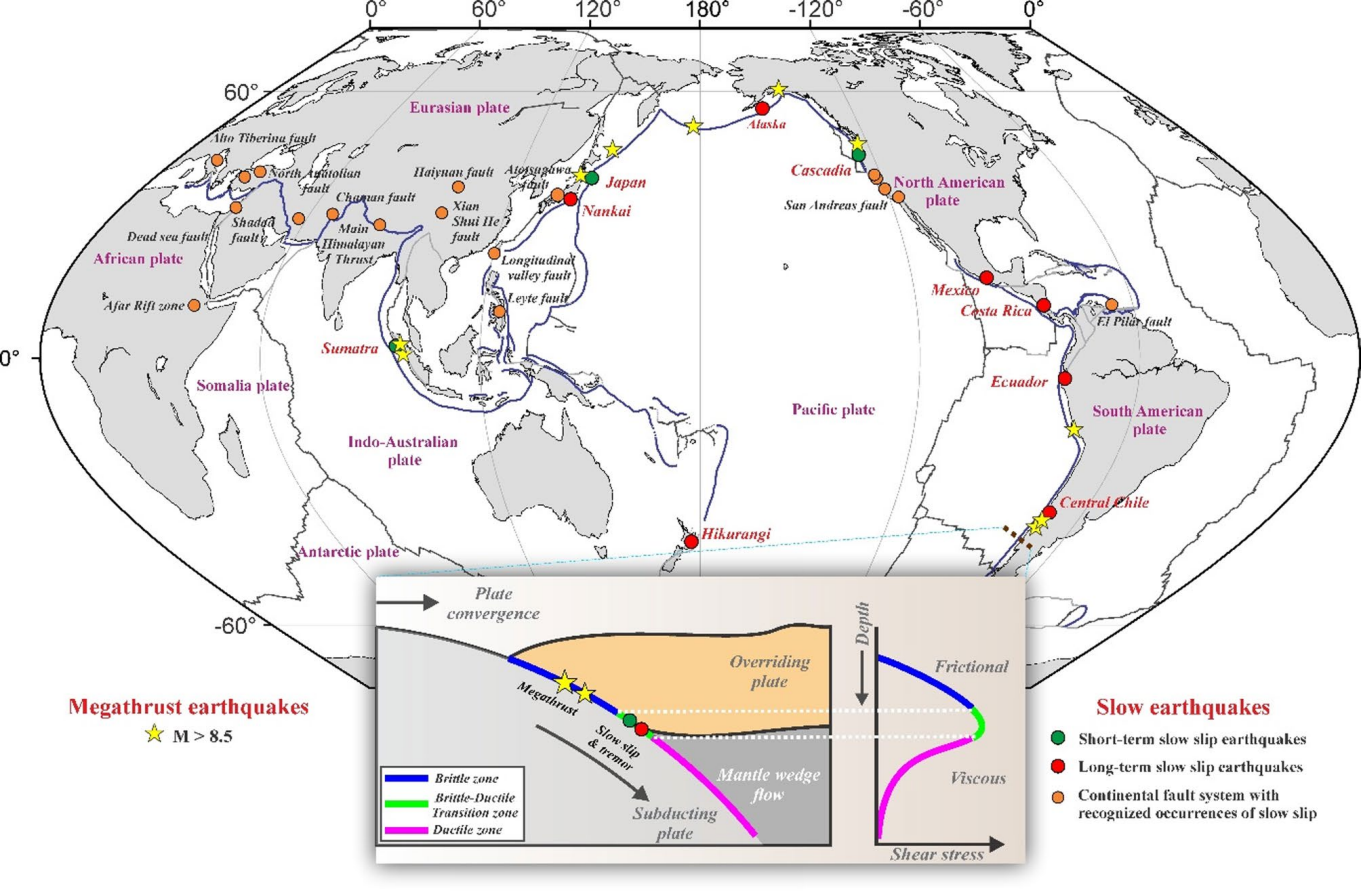
Distribution of slow earthquakes and megathrust earthquakes (M > 8.5) locations around the world. Yellow stars represent the megathrust earthquakes (M > 8.5), from USGS (https://www.usgs.gov/natural-hazards/earthquake-hazards/earthquakes). Green and red circles represent the short-term and long-term slow slip earthquakes, respectively (modified after Goswami and Barbot^12^; Obara & Kato^6^). Orange circles indicate the slow slip earthquakes on continental fault systems. (modified from Jolivet & Frank^9^). Blue and dark thin lines are subduction zones and active faults, respectively, archived from the Global Earthquake Model (http://globalquakemodel.org). The inset represents the cross-section of a subduction zone showing different segments of the fault along the depth of the subducting plate. Generic Mapping Tools (version 5.2.1; URL: https://www.soest.hawaii.edu/gmt/) and CorelDraw (version 18; URL: https://www.coreldraw.com/en/) have been used to generate this figure.

In contrast to the locked seismogenic zones, the down-dip segments of the subduction megathrust are often characterized by slow earthquakes (tremor and slow-slip events), which provide a wide spectrum of fault-slip behaviours and seismic patterns that are different from those of traditional up-dip megathrusts^6^although both share some common slip mechanism. Slow earthquakes, first discovered on the western coast of Japan^7^have since been detected globally, due to advancements in seismological and GNSS networks in recent decades^8^. They have been reported in diverse tectonic settings, particularly from the brittle-ductile transition zones along subduction megathrust (e.g., Japan, Cascadia, Mexico, Costa Rica, New Zealand and others)^9–11^ (Fig. 1) and are extremely sensitive to exogenous stress perturbations because of inherent fault weakness^9,11,13–17^. Notably, slow earthquakes, (including phenomena such as episodic tremor and slip (ETS)) are increasingly recognized as a key component of the slip budget at convergent plate boundaries, potentially mediating earthquake nucleation, rupture propagation, and arrest^6,9,10,18,19^. In fact, it has been proposed that slow earthquakes may act as a possible stress-meter, capturing time-dependent stress evolution on the adjacent up-dip seismogenic segments of the subduction megathrusts^6^. Factors like, (1) fault zone rheology, governed by mineralogy (e.g., serpentinite^12^) and grain-size evolution and (2) fluid-driven pore pressure variations^20^ and silica enrichment^21^ inducing a change in effective normal stress, dictate frictional stability and slip periodicity of the fault. Hence, studying the common slip mechanism of slow earthquakes, their interaction with neighbouring segments of the seismogenic zone, and the periodic occurrence of slow earthquakes may be useful for a better understanding of the earthquake physics underlying devastating megathrusts. In other words, slow earthquakes can be considered an important factor for studying megathrust earthquakes during great earthquake cycles in the convergent plate boundaries^6^. Thus, slow earthquakes (tremors, slow slips and ETS events) have become a focal point of scientific exploration and discussion in the geoscience community.

Slow earthquakes along subduction zones, typically recur quasi-periodically over months to years^6,22^ in contrast to megathrust earthquakes, which have recurrence intervals of centuries to millennia and release accumulated strain catastrophically. The limited span of modern seismic and geodetic records, along with the uncertainty of paleoseismic data or historical records, makes it difficult to fully characterize megathrust recurrence—a key component of long-term seismic hazard assessment and mitigation. In contrast, the high frequency and more accessible observational window of slow earthquakes offer an opportunity to study fault slip processes in real time. To address this gap, laboratory experiments and numerical simulations under the framework of rate-and-state friction laws have been routinely employed to replicate fault slip cycles under controlled conditions, generating synthetic datasets that capture multiple seismic and aseismic cycles—complementing sparse natural observations^23–25^. Despite these advances, a critical gap remains: how do slow earthquakes influence stress accumulation and release on adjacent megathrust segments? Specifically, can slow earthquakes act as reliable “stress-meters” for impending megathrust ruptures? Establishing a global parameter as a common linkage may allow us to indirectly infer the state of stress accumulation and the potential timing of future megathrust ruptures, particularly in regions where instrumental records are limited. Thus, investigating the recurrence characteristics of slow earthquakes and their relationship with megathrust events may provide a novel pathway to overcome the observational limitations posed by the long return periods of great subduction zone earthquakes. Growing evidence suggests a close spatiotemporal association of tremors (non-volcanic or tectonic i.e., which represent low-frequency seismic signals compared to regular fast earthquakes) with slow slip events^26,27^ thus, tremors can be considered as a direct indicator of the spatiotemporal evolution of slow slip events^9,10^.

Motivated by the above discussion, in the present article, we adopt a novel tripartite approach, using integrated constraints from (i) spatio temporal variations and recurrence intervals of SST from the two well-monitored subduction zones^28,29^ (i.e., Nankai subduction zone, western Japan and the Cascadia subduction zone, North America), (ii) numerical simulations of the slow earthquake cycle from a single-degree-of-freedom spring-block model under the framework of rate-and-state dependent frictional law, (iii) laboratory-based experimental results on stick-slip instability, and (iv) systematic comparison with global subduction zone datasets of megathrust earthquakes and slow earthquakes respectively, we have presented an interaction and a possible stress transfer from a slow earthquake source zone to the adjacent megathrust earthquake segments, as both of them share common slip mechanism. Finally, we argue that the slow earthquakes can be used as a proxy for the possible “stress-meters” for giant megathrust earthquakes nucleation processes.

The present article has been organised into the following sections. The datasets, numerical modelling strategy, and laboratory-based stick-slip experimental approach are presented in methods and supporting documents. Key findings related to down-dip/along-strike variation in SST events from two well-monitored subduction zones, along with the numerical model prediction of the natural geophysical observations, complementing with stick-slip experimental findings and systematic comparison with global subduction zone datasets are presented in the results section, before moving towards discussion, where we have presented robustness of our analysis and final concluding remarks. Further, all mathematical details and derivations, including dependency on various physical parameters, and tables of physical parameters have been presented in the supplementary information.

## Results

### Space-Time distribution of tremor and depth-dependent slip periodicity: cascadia vs. nankai subduction zone

**Fig. 2:**
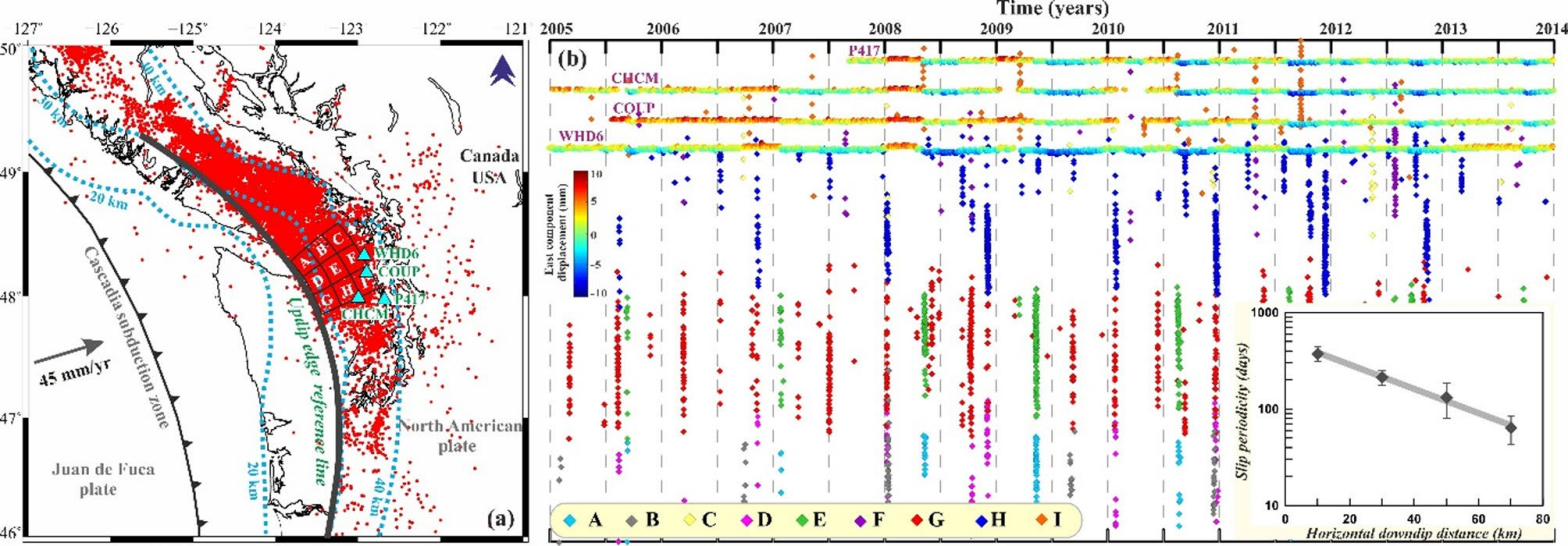
(**a**) Epicentral distribution of tremors in the northern Cascadia subduction zone for the period of 2005 to 2014 (red dots). Cyan dashed lines are the 20, 30, and 40 km slab isodepth. The up-dip edge of the tremor region is marked by the black line. The boxes (i.e., A, B, C, D, E, F, G, H, I) are the 20 km $\:\times\:$ 20 km tremor patches, which are further considered for slip periodicity analysis. Cyan triangles are the locations of the cGPS stations. (**b**) Spatio-temporal distribution of activity in tremor patches (marked by different colours) in the Cascadia subduction zone from 2005 to 2014. Inset (right bottom corner) shows the slip periodicity of the tremor patches as a function of the downdip distance of the fault from the up-dip edge of the tremor region (after Wech and Creager^29^). The top inset represents the displacement of the east component of the representative cGPS sites (shown in Fig. 2a). The colour bar represents the variation of the east displacement of cGPS sites as a function of time. The figures were generated using Generic Mapping Tools (version 5.2.1; URL: https://www.soest.hawaii.edu/gmt/), Grapher graphical application (version 8.7.844; URL: https://www.goldensoftware.com/products/grapher) and CorelDraw (version 18; URL: https://www.coreldraw.com/en/).

The Cascadia subduction zone is associated with the eastward oceanic Juan de Fuca plate subduction at the rate of ~ 40–45 mm/yr beneath the North American plate, from offshore northern California to Vancouver Islands, extending over 1000 km (Fig. 2a). The transition zone hosts repeated major ETS events in the northern section for ~ 3 weeks every ~ 15 ± 2 months^22,29–31^. Recent modelling studies of coupling^32,33^ and paleo-seismological observations^34,35^ indicate that coupling across the plate interface is sufficient to host giant megathrusts and resulted in past large earthquakes, the latest of which occurred on 26th January 1700, and recurrence intervals of large earthquakes of ~ 500–600 years over past ~ 8000 years.

**Fig. 3:**
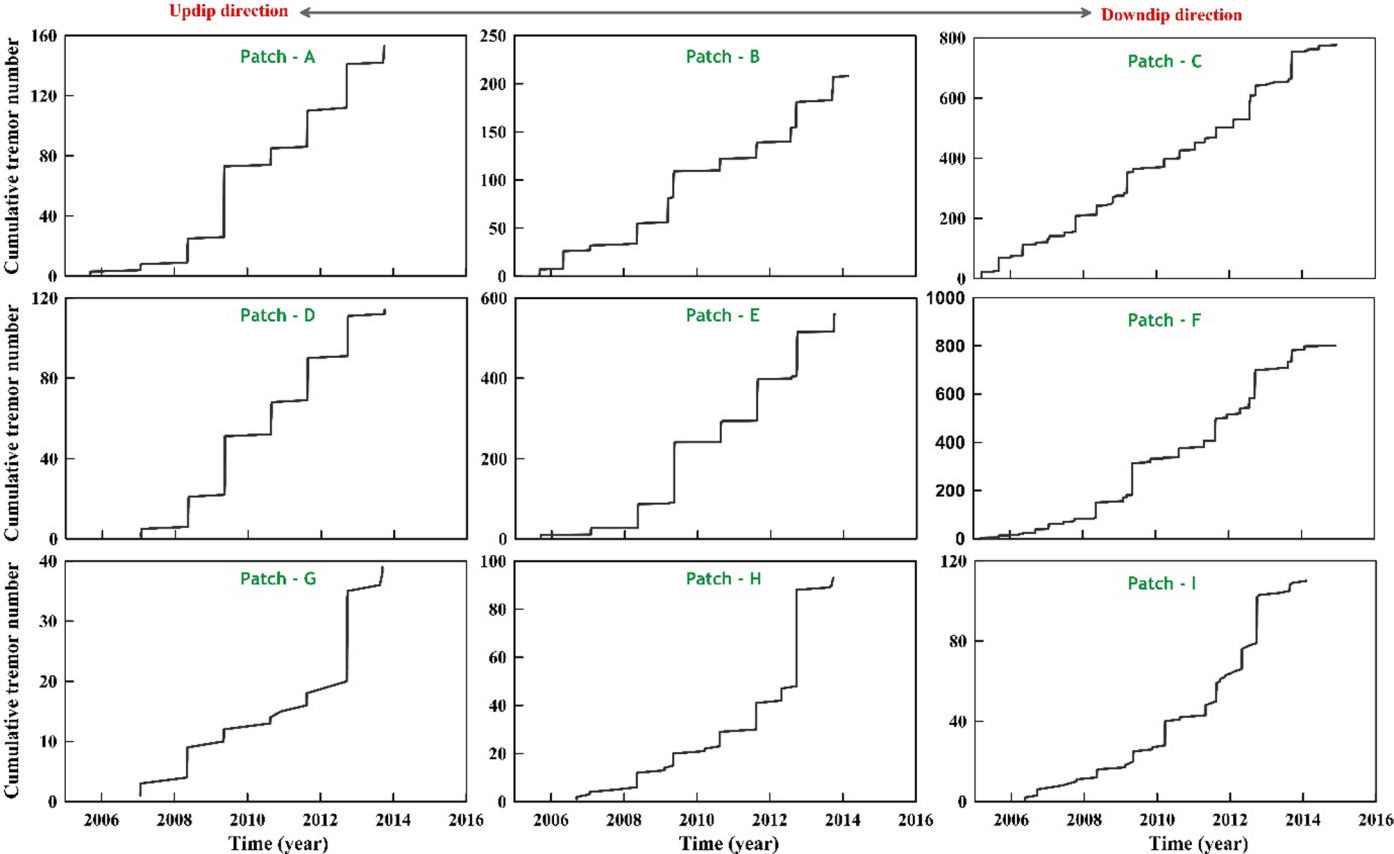
Variation of cumulative tremor number with time for all tremor patches (shown in Fig. 2a). Note that the tremor patches show a transition from the small frequent slip events in the down-dip direction to larger, less frequent slip episodes in the up-dip direction.

In Cascadia, the majority of tremors are located at the base of the seismogenic zone and where very-low-frequency (VLF) earthquakes occur. Here, we have re-examined the depth-dependent segmentation signatures in northern Washington (Fig. 2a), where the tremor region represents the widest, longest, and most homogeneous datasets of tremor occurrence to maximize the chances of resolving any depth dependency^29,36^. Figure 2b represents the space-time distribution of the tremor region along with the residual geodetic displacement time series (east), after removing the long-term plate motion of representative cGPS sites. We also present the distribution of the tremor region along with three profiles of cumulative tremor timelines in each, considering bins of 20 × 20 km^2^ across three strike-normal directions from the up-dip reference line of the transition region (i.e., total of nine bins A-I, Figs. 2b and 3). Moving from up-dip to downdip along three profiles in Fig. 3, a transition can be observed from the large and less frequent tremor episodes associated with geodetically resolved SST (i.e., represented by patches A, D, G) to small and frequent tremor activity (i.e., represented by patches C, F, I). Moreover, a decrease in relative shear strength from the up-dip to downdip direction is probable from this depth dependency of the slip periodicity (inset in Fig. 2b). In fact, from the average estimates of recurrence interval in each strike-normal bin, there is a roughly exponentially decreasing trend in slip periodicity with down-dip distance, which is consistent with the previous estimates^29^.

**Fig. 4:**
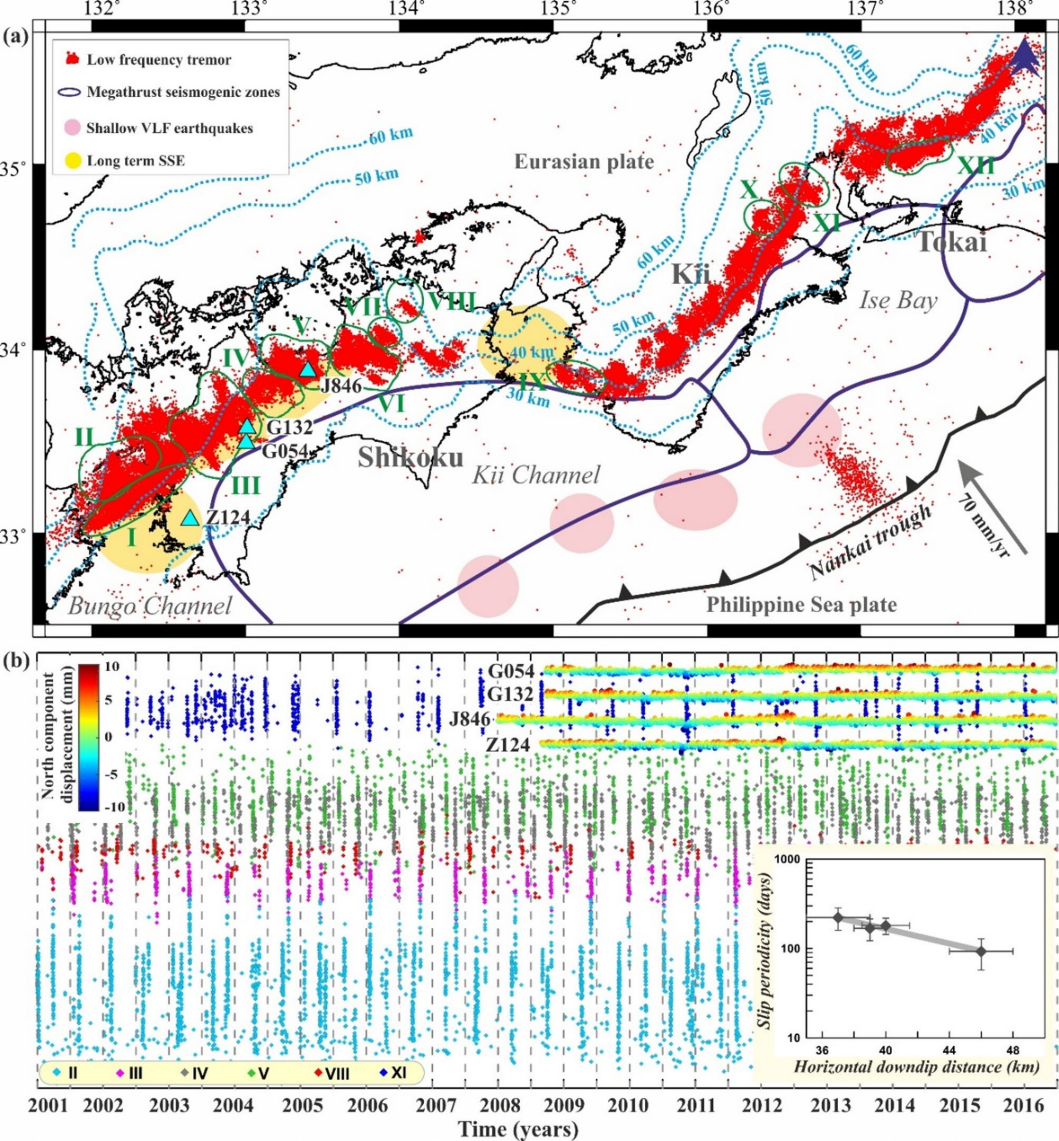
(**a**) Epicentral distribution of tremors in the Nankai subduction zone of South West Japan for the period of 2001 to 2016. Red dots are low-frequency tremors. Locations of long-term slow slip events (SSEs) and shallow very low-frequency earthquakes (VLF) are marked in the light pink and light yellow ellipses, respectively. The megathrust seismogenic zones along the Nankai subduction zone are marked in blue bold lines. Cyan dashed lines represent isodepth contours of the oceanic Moho discontinuity, derived from receiver function analysis (Shiomi et al.^46^), and different tremor patches are represented by green polygons. (**b**) Spatio-temporal distribution of tremors in the Nankai subduction zone for the period of 2001 to 2016. Inset (right bottom corner) shows the slip periodicity of the tremor patches (I, II, VII and IX) along the strike of the subduction zone as a function of downdip distance from the subduction zone. Top inset represents the displacement of the North component of the representative cGPS sites (shown in Fig. 4a). The figures were generated using Generic Mapping Tools (version 5.2.1; URL: https://www.soest.hawaii.edu/gmt/), Grapher graphical application (version 8.7.844; URL: https://www.goldensoftware.com/products/grapher) and CorelDraw (version 18; URL: https://www.coreldraw.com/en/).

In comparison with the Cascadia subduction zone, the occurrence of slow earthquakes, relative diversity, and spatiotemporal distribution in the Nankai Trough are relatively complex (Fig. 4a). The Nankai Trough formed by the subduction of the Philippine Sea plate beneath the Amurian microplate at a rate of ~ 65–70 mm/yr^37^. This subduction zone has been characterized by destructive megathrust earthquakes extending over ~ 1300 years^38^ and has hosted two recent megathrust earthquakes of M > 8 in the seismogenic zone in 1946 (M8.3 Nankai earthquake) and 1944 (M8.1 Tonankai earthquake) respectively^39,40^. Geodetically detected slow earthquakes are characterized by either long-term or short-term slow slip events with a duration ranging from years/months to days at the base of the seismogenic zone, whereas seismically-monitored shallow VLF earthquakes at the dominant period of tens of seconds have been observed within the shallow Nankai accretionary prism, and they likely occurred on the splay faults or the plate boundary decollement in the up-dip region of the megathrust/seismogenic zone^6,41–43^ (Fig. 4a). However, the deep tremor is distributed within narrow belt-like patchy regions along the down-dip edge of the megathrust/seismogenic zone. They continue for several days and are associated with both short-term slow slip events and deep VLF earthquakes^6,44^ (Fig. 4a).

**Fig. 5:**
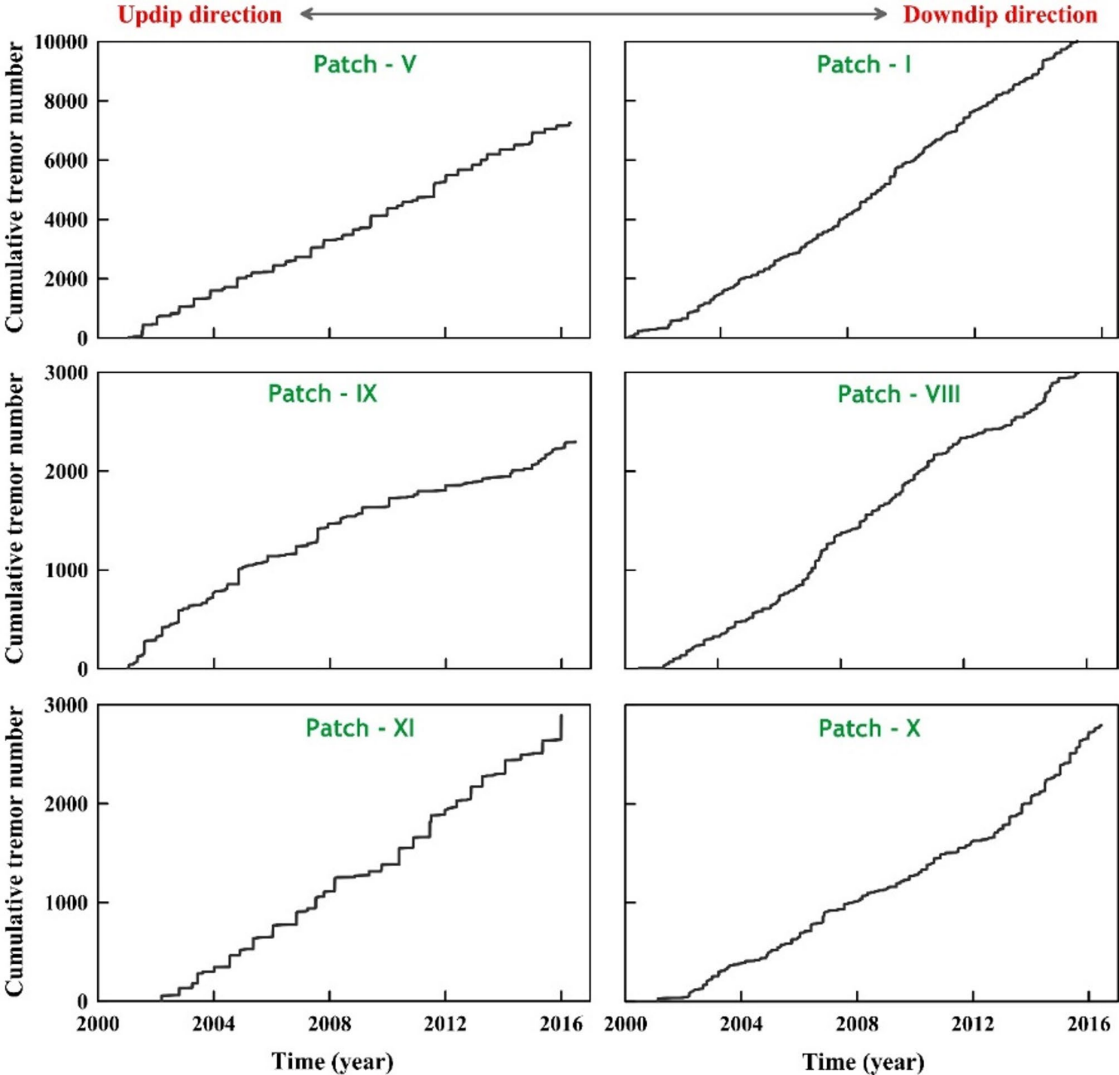
Variation of cumulative tremor number as a function of time for representative tremor patches (shown in Fig. 4a). Note that the tremor patches show a transition from larger less frequent slip events to smaller, more frequent slip events along the dip of the subduction zone (i.e., from updip to downdip direction).

Therefore, we suggest that these deep tremor patches can be considered as a direct proxy for the spatiotemporal evolution of the slow-slip events during ETS and the possible stress-transfer mechanism of the up-dip megathrust/seismogenic zone. This motivated us to explore the possible depth-dependent signatures of this narrow belt-like deep tremor (~ 600 km long and ~ 10–15 km wide), lying at a depth of 40–50 km on the subducted plate interface (Fig. 4a). We explore the possible relationship between the spatial distribution and size of the tremor burst. Figure 4b represents the space-time distribution of 12 selective patches of the tremor (marked as I-XII), which indicates variation in the tremor frequency and the cumulative number of tremor bursts (Fig. 5). Although tremor occurs overall as a narrow belt-like zone in the Nankai Trough, the distribution of the tremor source zones is extremely heterogeneous or patchy in nature (see some of the representative patches I-XII marked in Fig. 4a). It has been observed that, in north-eastern Kii and western Shikoku, the minor tremor activity has been located on the downdip side, whereas, in central Shikoku, tremor epicentres within shorter bursts are concentrated in the deeper side. However, in the eastern Shikoku, some isolated tremor clusters, apart from the belt-like tremor zone, are composed predominantly of minor tremor bursts^45^.

Figure 5 represents the cumulative number of tremor activities in representative patches along the dip of the subduction zone, (Figure S11, all patches from I to XII). From this plot (Fig. 5), we observe typical step-like cumulative curves for some groups on the up-dip edge (Patch V, IX, XI). This indicates possibly active tremor bursts containing many tremor events that have occurred in regular intervals. However, on the down-dip edge (Patch I, VIII, X), the tremor activities are relatively gradual, with frequent occurrences of minor tremor bursts containing small numbers of tremor events. Although the width of the tremor region at the Nankai Trough is relatively narrow compared to the Cascadia subduction zone in northern Washington, hence the variation is not as apparent as in Cascadia nevertheless, a decreasing trend in the slip periodicity with down-dip distance appears in both well-monitored subduction zones (inset in Figs. 2b and 4b). These observational results highlight a systematic depth-dependent pattern in tremor recurrence intervals; next we investigate the role of general kinematic factor like plate motion or loading rate which may govern slip periodicity. To isolate the role of V_L_​, we next employ numerical simulations under controlled rate-and-state friction (RSF) parameters.

### Numerical simulation of depth-dependent slip periodicity

**Fig. 6:**
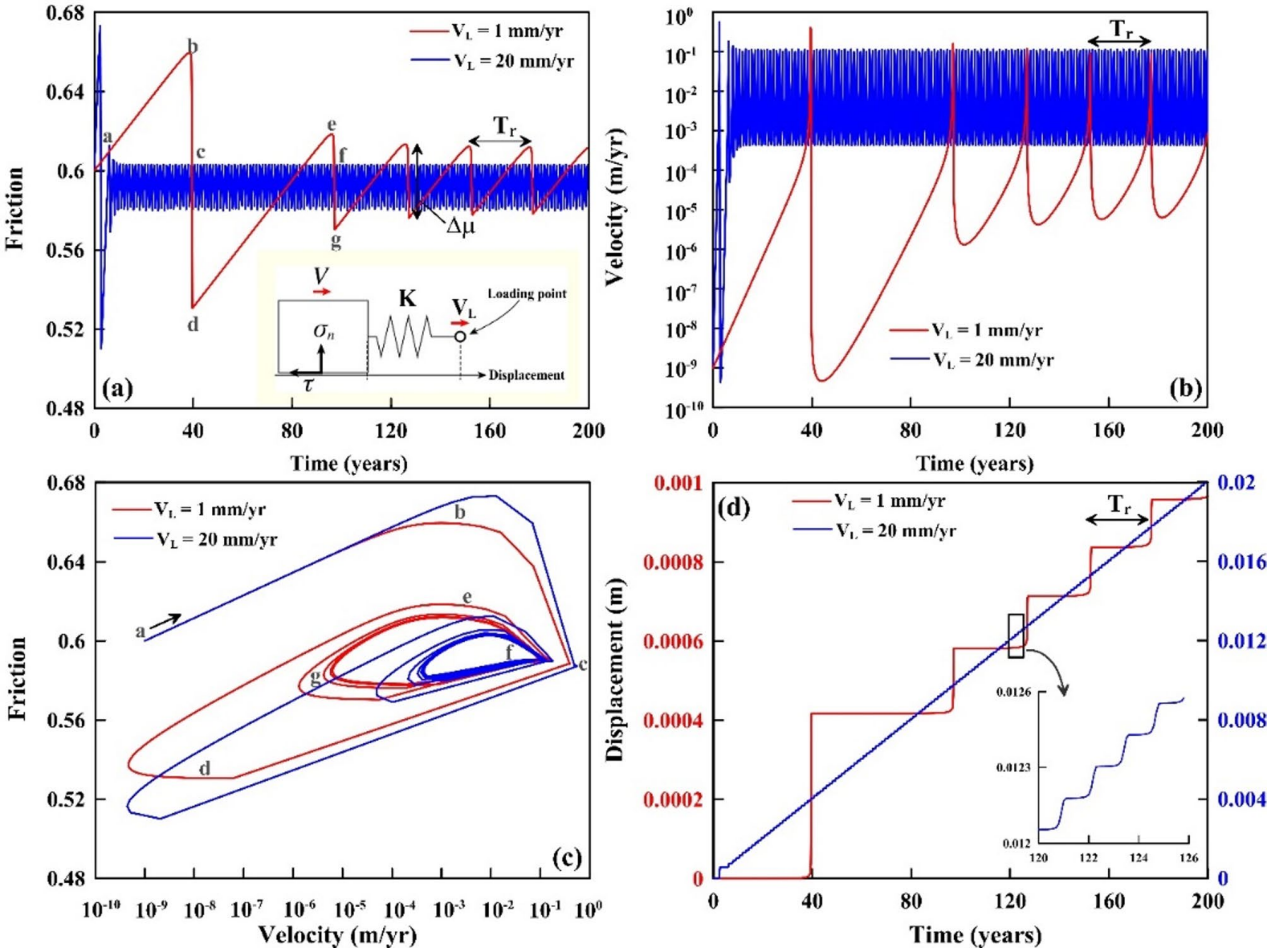
Simulation of stick-slip motion. (**a**) Friction response, (**b**) velocity response, (**c**) phase plane plot (friction-velocity), and (**d**) displacement during stick-slip motion considering ageing law. The red and blue lines represent loading velocities of 1 mm/yr and 20 mm/yr, respectively. Inset (in panel a) is the schematic representation of the spring-slider system, where V_L_ is the loading velocity, K is spring stiffness, σ_n_ is normal stress, τ is the shear stress, and V is the velocity of block. Inset (in panel d) is the zoom version of the displacement variation during the stick-slip motion (rectangle in Fig. 6d). Note that, the recurrence time (T_r_) and friction drop (µ) increases with decreasing the loading velocity.

We perform a series of systematic numerical simulations using the approach outlined in the methods section (along with mathematical details and derivations in supporting documents) to represent the healing effect on the prescribed rate-and-state frictional parameters a, b, L and the loading velocity V_L_ (Fig. 6a). Figure 6 shows representative quasi-dynamic simulation results to demonstrate the evolution of friction response (Fig. 6a), velocity response (Fig. 6b), phase diagram (friction-velocity) (Fig. 6c), and displacement response (Fig. 6d) during stick-slip motion. In the numerical simulations, we assumed the state evolution ageing law with the input parameters: $\:{\mu\:}_{0}$= 0.6, $\:{V}_{*}$ =10^− 9^ m/s as an arbitrary reference velocity, a = 0.005, b = 0.008, L = 5 μm, $\:{\sigma\:}_{n}$ = 10 MPa, along with a systematic variation for the two loading velocities V_L_ (1 mm/yr and 20 mm/yr). To reproduce unstable stick-slip motion in the system, we consider (a – b) < 0 and K < Kc, where Kc represents the critical stiffness of the spring that determines the slip stability.

The Rate and State Friction (RSF) model, which describes how frictional strength evolves with sliding rate and time, operates within specific physical constraints such as inertia (or mass), damping coefficient, temperature, and subduction zone fault dip, all of which can influence the numerical simulation model’s outcomes. We tested these factors individually (details in supporting information) and found that while the mass (inertia) of the block, damping coefficient, and dip of the frictional surface change the slip velocity and shear stress, they do not affect the dynamic stability of the system; temperature influences stability dynamics indirectly through shear heating, while critical slip distance and normal stress have minimal but notable effects on slip recurrence and amplitude. This confirms their minimal impact on the overall outcomes of the analysis. Therefore, we have conducted the study while neglecting these factors, as they do not significantly influence the system’s stability.

From Fig. 6, it can be observed that all numerical simulations initially demonstrate a large stress drop in response to strong healing, however, that larger initial stress drop gradually decreases with the progress of the stick-slip sequence, and after that, it becomes periodic (Fig. 6). A longer recurrence time (T_r_) is associated with greater friction drop (or static stress drop Δµ) with a typical logarithmic dependency. The recurrence time (T_r_) of the stick-slip motion is further inversely related to the loading velocity V_L_, in other words, a longer T_r_ is associated with a smaller V_L_ with a logarithmic dependency (Fig. 6).

**Fig. 7:**
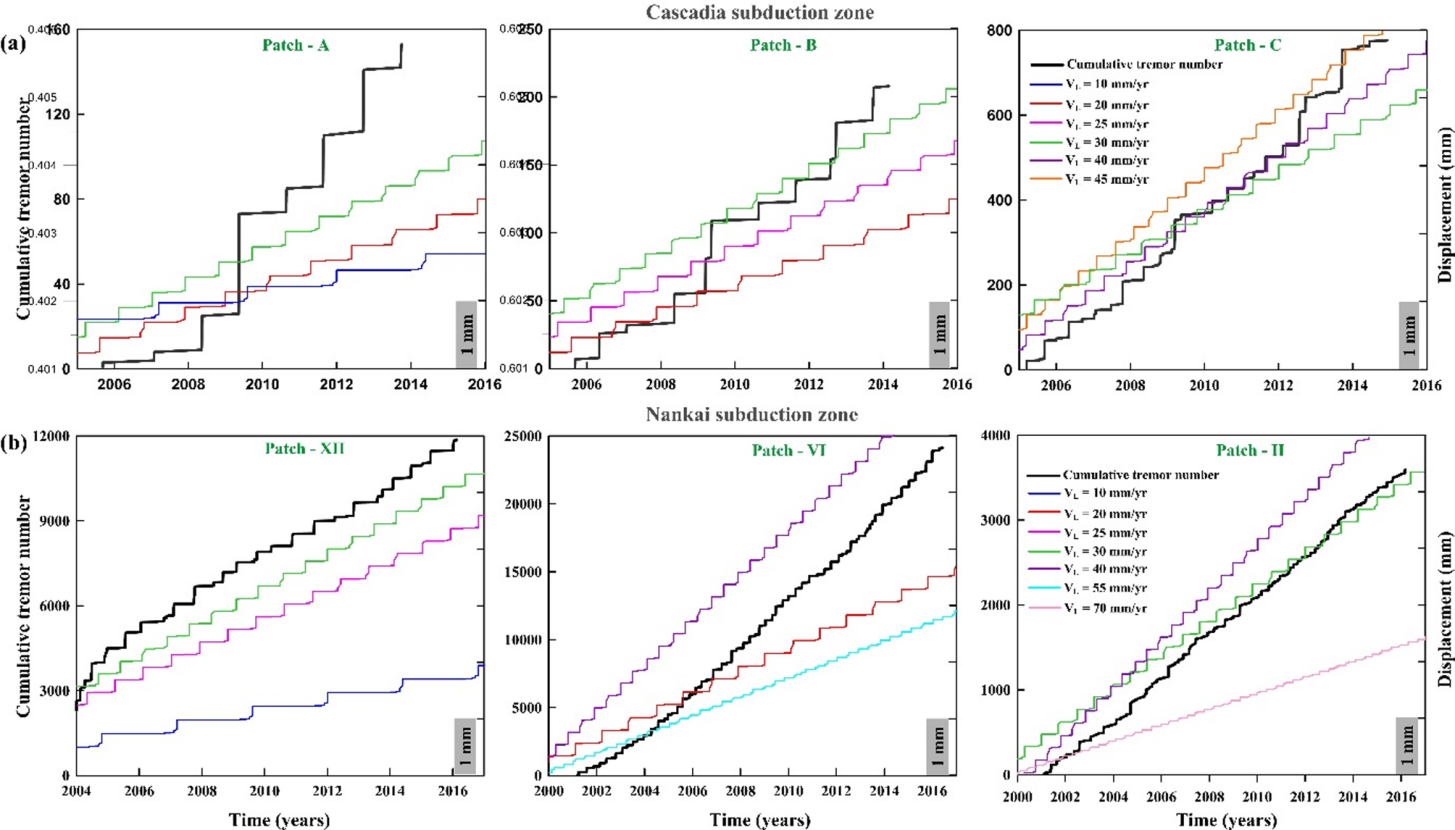
(**a**) Variation of cumulative tremor number (black line) with time for representative tremor patches of the Cascadia subduction zone (shown in Fig. 2a). The simulated displacement response for various loading velocities during the stick-slip motion is represented by the different colour lines. Note that the slip of the tremor patches in up-dip region is matched with the model displacement curve of low loading velocity, whereas the slip of the tremor in the downdip portion of the tremor zone is matched with the model displacement curve at higher loading velocities. (**b**) Same as Fig. 7a but for the Nankai subduction zone.

Moreover, we suggest that this inverse logarithmic dependency between recurrence time (T_r_) and loading velocity (V_L_) of the stick-slip motion appears to be consistent with the depth dependency of the tremor activities and the associated slip-periodicity behaviours that have been observed in the space-time distribution of tremor in Cascadia and Nankai subduction zones (Figs. 2, 3, 4 and 5). Depth-dependent variations in fault coupling are widely observed in subduction zones and are consistent with geodetic inversions^32,33^. These variations reflect how subduction interfaces accommodate plate motion differently with depth, often transitioning from locked to creeping behavior downdip. In our numerical simulations (Fig. 7a and b), we clarify that velocity loading (V_L_) qualitatively represents the localized rate of slip accumulation along the downdip megathrust, rather than the overall plate convergence rate. This interpretation is consistent with the notion that V_L_ variations capture spatial changes in fault coupling inferred from geodetic studies^32,33^. For example, Cascadia’s downdip tremor zone accommodates 80–90% of plate motion, while the locked zone accumulates slip at ~ 5–10 mm/yr^32^.

To match the observed space-time tremor distribution of the different patches in the Cascadia and Nankai subduction zone, we also present model predicated displacement from the spring-block model, considering different loading velocity (V_L_) (Fig. 7). In this study, we concentrate on the recurrence times (Fig. 7) comparing both numerical simulations (coloured curves of different velocities) and observed tremor variations. The variations in cumulative tremor (black curve) and displacement (coloured curves from simulation at different velocities), represented on the y-axis, are not a significant concern, as these parameters are expected to be uncorrelated. Cumulative tremor counts are commonly used as a proxy for slow slip displacement, based on empirical correlations in subduction zones like Cascadia and Nankai, where tremor duration scales linearly with geodetically measured slip^26,27^. Though not a direct measure, tremor counts offer a first-order estimate of slip progression in regions lacking continuous geodetic data. They also help map along-strike variations in slip behavior, effectively linking tremor activity to very low-frequency (VLF) slip gradients^6^. We observed that the slip distribution pattern of tremors in the up-dip direction (Patch A) of the Cascadia subduction zone matches well with the model predicted recurrence interval corresponding to lower loading velocity ~ 20 mm/yr while in the down-dip direction (Patch C) it matches the model-predicted recurrence interval corresponding to higher loading velocity ~ 45 mm/yr (Fig. 7a). Interestingly, this velocity never exceeds the velocity of down-dip plate motion for the Cascadia subduction zone even at the deepest segments of the tremor patch just above the creeping segments. This observation is also consistent with the numerical simulations for the Nankai subduction zone (Fig. 7b). We argue that such unique consistency demonstrates a complex subduction plate kinematics or variation in kinematic coupling along the infinite segments of laterally connecting frictional patches, from the up-dip seismogenic megathrust faults to down-dip slow earthquake segments. The depth-dependent slip periodicity observed in Cascadia and Nankai arises from transitions in frictional stability governed by the rate-and-state parameters a, b, and L. At greater depths, elevated temperatures and pore pressures reduce effective normal stress (*σ*_*eff*_​​), promoting velocity-strengthening behavior (a − b > 0) and frequent slow slip events^21,47^. Our simulations replicate this behavior by varying V_L_ (a proxy for plate convergence rates): higher V_L_ downdip shortens recurrence intervals (Fig. 7), consistent with tremor frequency increasing with depth (Figs. 2, 3, 4 and 5). Therefore, different loading velocities at different depths indicate its control over the recurrence interval from the simulation approach. Next, we investigate this role of V_L_ as a controlling factor over recurrence interval, through laboratory experiments by systematically increasing the loading velocity in each experiment.

### Comparisons with laboratory-based experimental observation

**Fig. 8:**
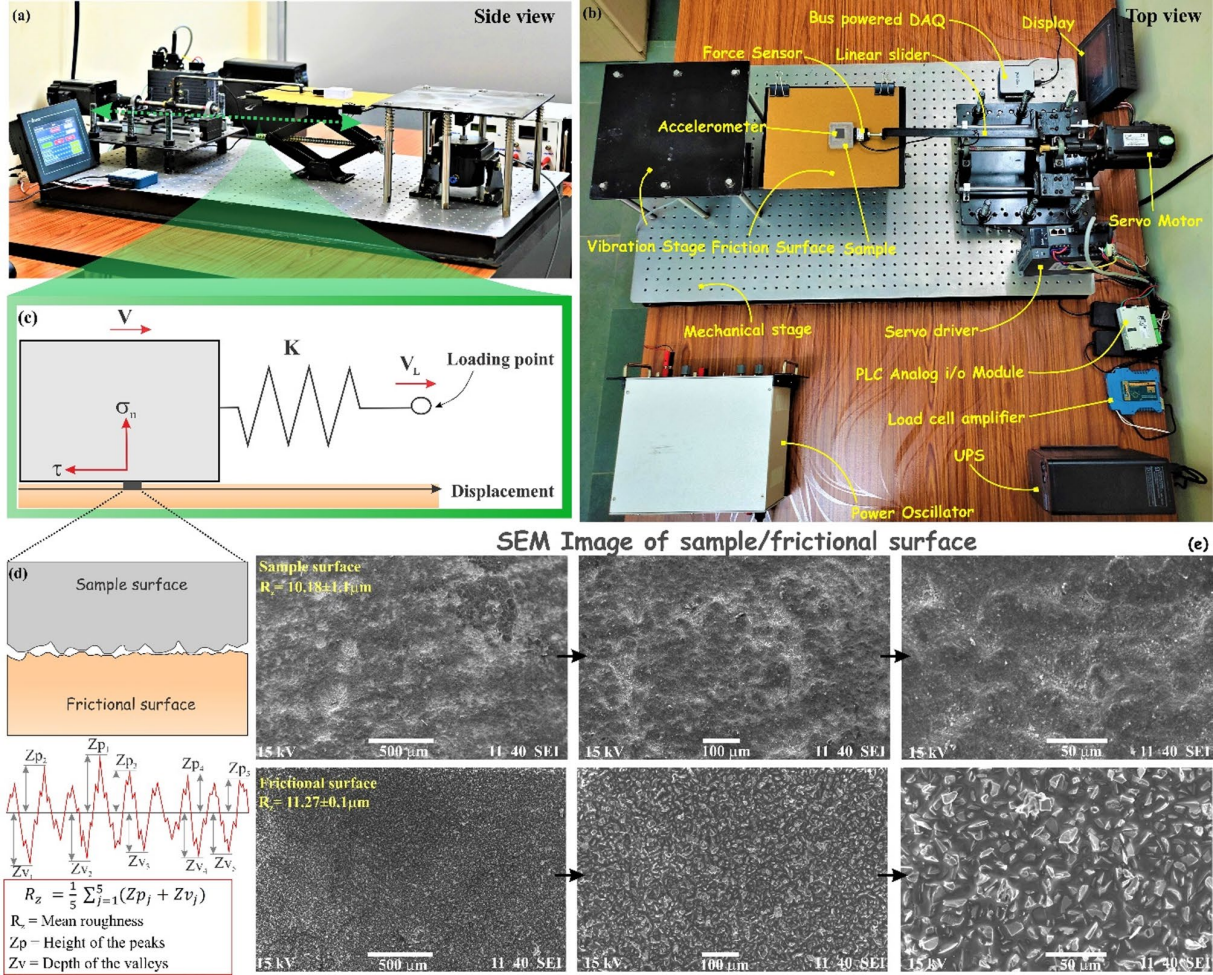
(**a**, **b**) Top and side view of the experimental setup labelled with its different components. (**c**) The schematic representation of a spring-slider system is analogous to the experimental setup. A block is attached to the spring having stiffness K and pulling with a constant velocity$\:{\:V}_{L}$ against a flat surface. The block generates frictional resistance force τ when the block moves with a velocity of V. (**d**) schematically represented contact area between the sample and the frictional surface. The parameter (R_z_) characterises the roughness of the surface, which is the average value of the heights of the five highest-profile peaks and the depths of five deepest valleys. (**e**) Scanning electron microscopy (SEM) images of the sample (top panel) and the frictional surface (bottom panel) under three different magnifications, represented by three different scales 500 μm, 100 μm, and 50 μm from left to right, respectively.

To understand the influence of loading velocity on the stick-slip/earthquake cycles and frictional strength evolution of fault, we have performed the stick-slip experiments considering a single degree of freedom spring-block model by varying the loading velocity over a range of 2–100 μm/s at constant normal stress (Fig. 8). The estimated mean roughness (R_z_) for the sample surface (i.e., sliding block) and the frictional surface is about 10.18 ± 1.1 μm and 11.27 ± 0.1 μm, respectively which we kept constant throughout all experiments, and the other parameters are shown in supplementary Table T1. During the experiments, we measured the force drop and acceleration of each stick-slip experiment for a specific loading velocity.

**Fig. 9:**
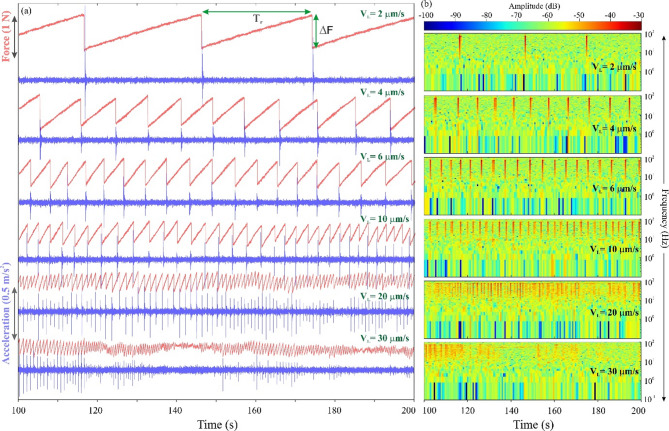
(**a**) Variation of force drop and acceleration as a function of time for different loading velocities observed during stick-slip experiments. (**b**) Frequency spectrum analysis for acceleration data of the respective force drop time series generated from the laboratory base experiments.

Figure 9a represents the variation of force change and acceleration as a function of time for a few representative stick-slip experiments. It can be noted that each earthquake cycle starts with a gradual build-up of stress called the stick phase when the static friction is greater than the dynamic friction, followed by an instantaneous rapid acceleration of the sliding block on the frictional surface, known as the slip phase (Fig. 9a). From this laboratory-based experiment result, it has been observed that the number of the stick-slip/earthquake cycles increase with a systematic increase in the loading velocity. Further, we have analyzed the frequency spectrum of the acceleration data of the respective force drop time series generated during all stick-slip experiments, which helps us to isolate the noise from the signal. A representative frequency spectrum is presented in Fig. 9b. From this analysis, we observe that the high energy peaks are characterised by the amplitude and reaching frequency peaks of ~ 10–100 Hz, which coincide with the co-seismic slip phase (i.e., peaks of the force drop and/or acceleration), confirming that the observed slips are seismic in nature (Fig. 9b). The slip phases are isolated by the background noise, which is of lower amplitude and high frequency. Slip phases are also interspersed between small amplitude lower frequency peaks (i.e., below 1 Hz) (Fig. 9b). These small amplitude lower frequency peaks in the frequency spectrum possibly represent slow slip or post-seismic after-slip events (Fig. 9b).

**Fig. 10:**
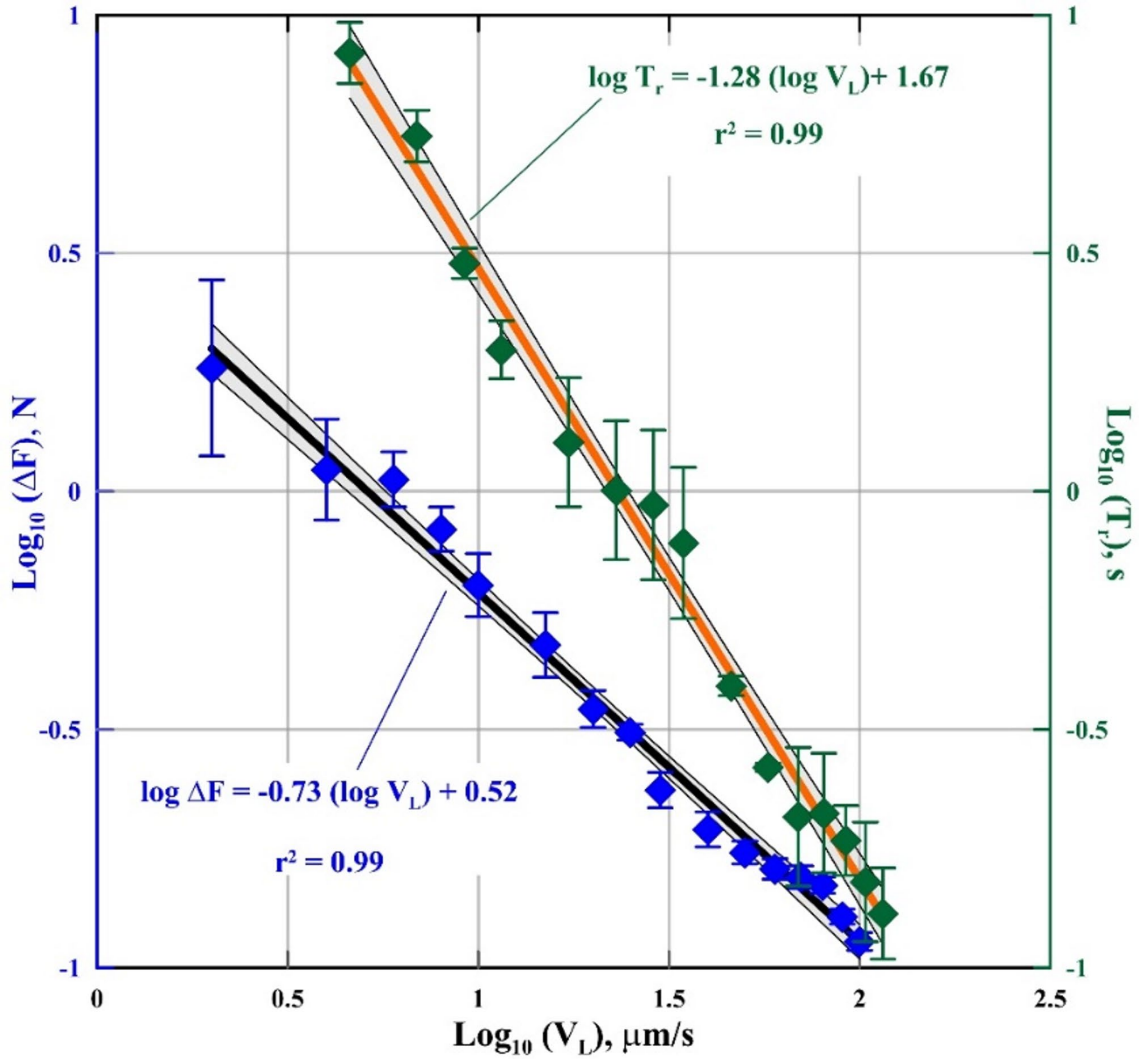
Variation of force drop (*black line*) and recurrence time (*orange line*) as functions of the loading velocity, plotted in a log-log scale. The gray shaded region marks the area under 95% confidence interval. The force drop and recurrence time are inversely related to the loading velocity.

Im et al.^25^ from their laboratory data on stick-slip motion also suggested that static stress drop (Δµ) had a logarithmic dependence on event recurrence time T_r_ in periodic motion^48–51^. We quantified the force drop (ΔF) and recurrence time (T_r_) (i.e., the time between two successive stick-slip events) for each loading velocity experiment and plotted their respective means as a function of the loading velocity in a log-log scale (Fig. 10). The best fit lines as illustrated in the Fig. 10 yield the following two equations:

1 $$\:\left.\begin{array}{c}log\:{T}_{r}=-1.28\text{log}{V}_{L}+1.67\\\:\text{log}\varDelta\:F=-0.73\text{log}{V}_{L}+0.52\end{array}\right\}$$

Combining the above equations we get:

2 $$\:\text{log}{T}_{r}=1.75\text{log}\varDelta\:F+0.75$$

**Fig. 11:**
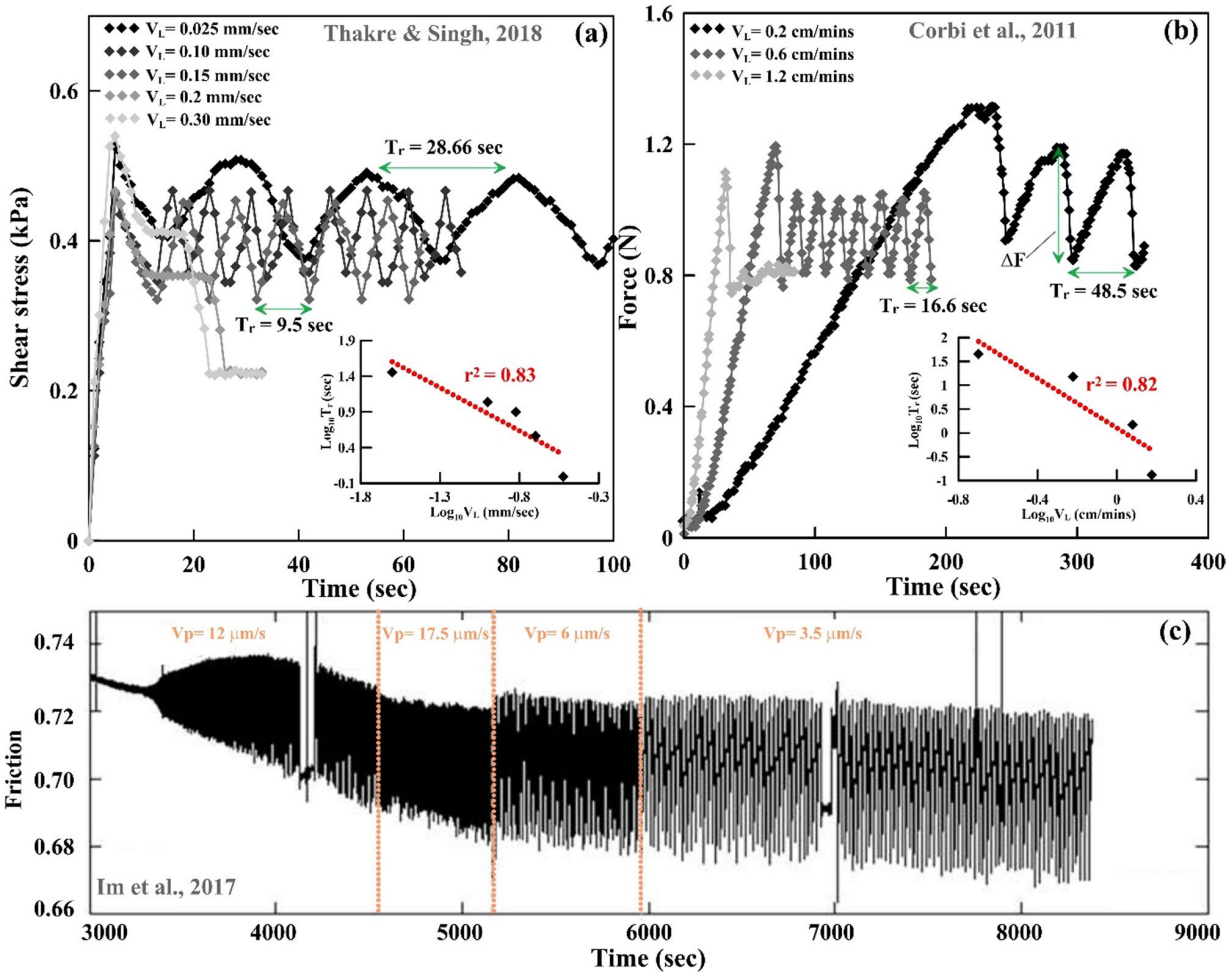
Variations of friction, force, and shear stress are plotted as functions of time, for different loading velocity. Note that the recurrence time is inversely related to the loading velocity (inset), which is consistent with our laboratory experiment results (modified after Im et al.^25^; Thakre and Singh^52^; Corbi et al.^53^).

Therefore, our laboratory experiments (not scaled) show (T_r_ varies as a function of Force drop) a relationship exists between Force drop (∆F) and recurrence interval. Future studies could greatly benefit from exploring the relationship between force drop and recurrence time in real-world earthquakes using scaled experiments. This area of research holds significant potential for advancing our understanding of seismic behaviour. However, the challenge lies in the scarcity of continuous stress drop (or force drop) time series datasets, along with other unpredictable factors, which complicates the feasibility of such analyses. Addressing these data limitations will be crucial for more comprehensive investigations in this field. More importantly, we observe that both the recurrence time and force drop decrease logarithmically with an increase in the loading velocity (Fig. 10), indicating that with an increase in the loading velocity, the system may change from the stick-slip domain to the stable slip domain. This laboratory-based observation is also consistent with the numerical model simulations and with the observations of depth-dependent slip periodicity and stress drop (presented in the results and Fig. 7). We compared our laboratory results with previous investigations^25,52,53^ and we noticed that our laboratory-based experimental results are aligned with, and provide support for, those findings (Fig. 11). Laboratory results confirm that recurrence intervals (T_r_) scale logarithmically with loading velocity (V_L_). Next, we investigate whether such a relationship exists in natural observations across major subduction zones around the Pacific Ring of Fire.

### Slow-earthquake periodicity and recurrence time of megathrust earthquakes

**Fig. 12:**
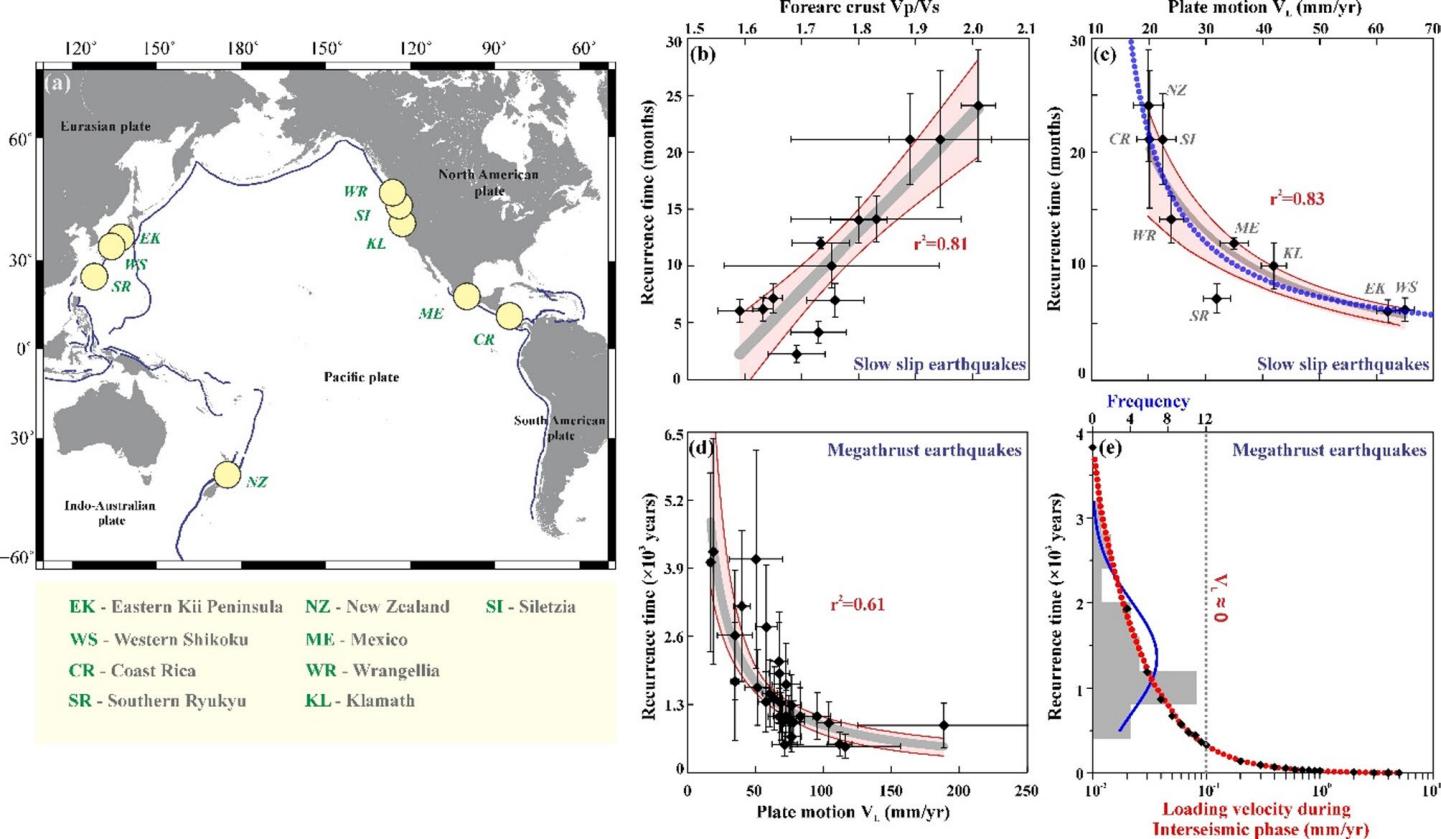
(**a**) Global distribution of slow slip earthquakes in subduction zones. The orange-shaded region marks the area under a 99% confidence interval in the following figures. (**b**) Recurrence time of the slow slip earthquakes as a function of Vp/Vs of the overlying forearc crust. (modified from Audet & Burgmann^21^). (**c**) The recurrence time of the slow slip earthquakes (from the subduction zones marked in Fig. 12a) as a function of plate convergence rate (grey curve). The overlapping blue curve represents the variation of the model simulated recurrence time with plate motion for low normal stress (~ 0.1 MPa). The recurrence time increases exponentially with a decrease in relative plate velocity (**d**) Variation of recurrence time of megathrust earthquake with plate motion. (**e**) Histogram of the recurrence time of megathrust earthquakes and variation of model simulated recurrence time with plate motion (red curve). The figures were generated using Generic Mapping Tools (version 5.2.1; URL: https://www.soest.hawaii.edu/gmt/), Grapher graphical application (version 8.7.844; URL: https://www.goldensoftware.com/products/grapher) and CorelDraw (version 18; URL: https://www.coreldraw.com/en/).

In the past two decades, along with megathrust earthquakes, slow earthquakes have been detected through seismic and geodetic instruments in the global subduction zones^6,54,55^. In Fig. 12 (a), the map shows the subduction zone boundaries (thin purple lines) with selected regions marked (yellow circles) from where the recurrence time data of slow slip and megathrust earthquakes have been obtained for the analysis. Here, we present a compilation of seismic data^21^ from these regions exhibiting recurring slow earthquakes as a function of the average ratio of P-wave (compressional) velocity to S-wave (shear) velocity (Vp/Vs) for the overlying forearc crust (Fig. 12b). As has been reported in the literature^51^ the overriding forearc crust’s Vp/Vs ranges between 1.6 and 2.0 and shows a linear and positive relationship (r^2^ = 0.81) with the average recurrence time of slow earthquakes. This result supports the hypothesis that the Episodic Tremor and Slow slip (ETS) behaviour is determined by the structure of the forearcs in the hanging wall of the subduction zone. The low forearc Vp/Vs indicate a progressive silica enrichment with depth^21^. The factors that control the ETS periodicity are poorly understood. The decrease in the recurrence time of tremor activity with progressive silica enrichment (i.e., low Vp/Vs value with depth) was explained^21^ as a consequence of temperature-dependent fluid pressurization and reduction in healing and permeability of fault gauge via dissolution-precipitation creep^56^. Numerical models of slip stabilization have also shown that an increase in dilatancy increases the recurrence times and slip amplitude of slow slip events^57^.

For constant low effective normal stress as an input parameter, our numerical modelling result (blue curve) shows a strong correlation (r^2^ = 0.88) between the recurrence time and the loading velocity (Fig. 12c) which probably indicates the presence of anomalous fluid. This presence of high-pressure fluid at the depth of the tremor causes a rapid reduction in the effective fault normal stress and more frequent slip events^21^. While scatter in global T_r_-V_L_​ data (Fig. 12b) has been attributed to lithospheric heterogeneity that modulates the effective normal stress, our simulation of slow earthquakes (Fig. 12c, blue curve) simplifies effective normal stress (*σ*_*eff*_) as constant. However, we acknowledge that spatial variations in (*σ*_*eff*_) driven by fluid pressurization (*P*_*p*_)^21,58^ contribute to SSE periodicity. For instance, elevated (*P*_*p*_) in high Vp/Vs regions reduces (*σ*_*eff*_), promoting frequent SSEs.

Note that the recurrence time from the numerical model increases exponentially with a decrease in the loading velocity (analogous to the plate motion for slow slip earthquakes from the natural catalogue data). Therefore, our numerical modelling result for slow earthquakes (overlapping blue curve, Fig. 12c) under constant effective normal stress seems to complement the natural observations (grey curve), i.e., an exponential decrease in the recurrence time of slow earthquakes and similar characteristic is observed for megathrust earthquakes (Fig. 12d). Further, we also notice that the recurrence times for the megathrust earthquakes are at intervals of more than 1000s of years, as the loading velocities during the interseismic phase must be closer to zero, as is evident from the intersection of loading velocity (red curve) with the histograms indicating the frequency of occurrence of megathrust earthquakes (Fig. 12e). Although the recurrence time of megathrust earthquakes decreases logarithmically with their respective plate velocity, their correlation is very weak (r^2^ = 0.64).

**Fig. 13:**
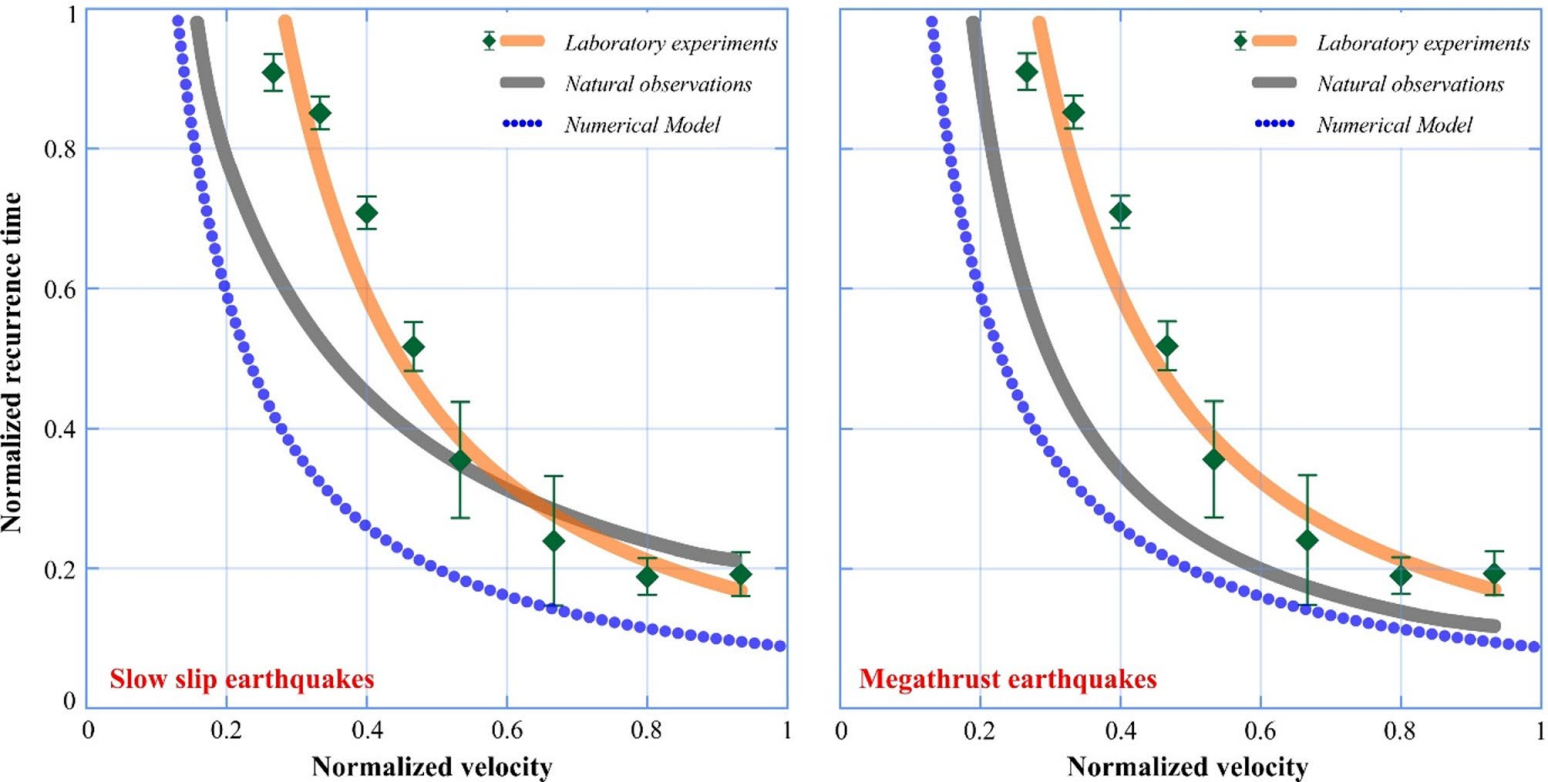
Normalized velocities as a function of normalized recurrence time for slow slip events and megathrust earthquakes. The blue curve represents results of numerical model; the orange and grey curves indicate results of natural observations and laboratory experiments, respectively.

We compare the relation between the recurrence time and plate motion/loading velocity from natural observation, numerical modelling, and laboratory experiments using the normalization technique (Fig. 13). The normalization is used to make these datasets dimensionless and in the same range for better understanding and comparison of results. The data points of each parameter are divided by a value close to the upper limit. For example, the recurrence times of megathrust earthquakes and slow slip events are normalized by dividing the data by an upper limit value of 5000 years and 25 months, respectively. Similarly, the plate velocities of megathrust earthquakes and slow slip events are normalized by 150 mm/yr and 75 mm/yr, respectively. To compare the natural observation results with the findings from our laboratory experiments (where we see logarithmic scaling of T_r_ with V_L_) and numerical modelling, we normalized the recurrence times of stick-slip events from laboratory experiments by 1.1 s and from numerical modelling by 15 months while the loading velocities are normalized by 75 μm/s and 100 mm/yr, respectively. In Fig. 12, we present the normalized recurrence times of megathrust earthquakes and slow slip events from real-world data (in grey) as a function of normalized plate velocity in the respective regions. Comparing laboratory experiment data (in orange) of repetitive stick-slip events where the normalized repeat intervals and loading velocities are analogous to the recurrence times and plate velocities of the real-world data, we observe a high correlation between the two parameters for both natural data and numerical modelling result. The striking similarity of the curve fit for the laboratory data and numerical simulation results with the natural observations reveals that our results closely mimic the natural phenomena and can be modelled by using/assuming a simple single-degree of freedom spring-slider experiment. This asserts that the logarithmic scaling of T_r_ with V_L_ (Fig. 12c) persists globally as well, across subduction zones with varying *σ*_*eff*_, suggesting that plate kinematics imposes a primary control. To our knowledge, this is the first experimental stick-slip cycle demonstration of logarithmic T_r_-V_L_ scaling with natural observations on a global scale (Fig. 13), thereby, bridging the scale gap between mm-sized lab faults and km-scale subduction zones. This aligns with laboratory studies where T_r_ scales with V_L_ even under variable effective normal stress^25^. Future work will integrate *σ*_*eff*_ -dependent RSF laws to refine these dynamics.

Dal Zilio et al.^59^ demonstrated that faster convergence cools the megathrust, expanding seismogenic zones and increasing large earthquake frequency. Such mechanisms may explain deviations in SSE recurrence (e.g., scatter in Fig. 12b) and transient stress transfer efficiency. For instance, high fluid pressure in the Nankai Trough^60^ could enhance SSE-megathrust coupling, whereas low permeability in drier margins (e.g., Chile) may decouple these processes. While Dal Zilio et al.^59^ attribute seismicity to thermal controls, the stress-meter hypothesis posits that SSEs dynamically regulate stress thresholds. Our results complement previous studies by demonstrating that slip velocity (*V*_*L*_) also influences SSE recurrence (Fig. 12c), potentially preconditioning megathrust rupture. However, our model assumes a constant effective normal stress and does not account for fluid-driven processes such as permeability evolution and episodic fluid pressurization^20^. Future models integrating hydromechanical feedback with rate-and-state friction could refine the stress-meter hypothesis.

**Fig. 14:**
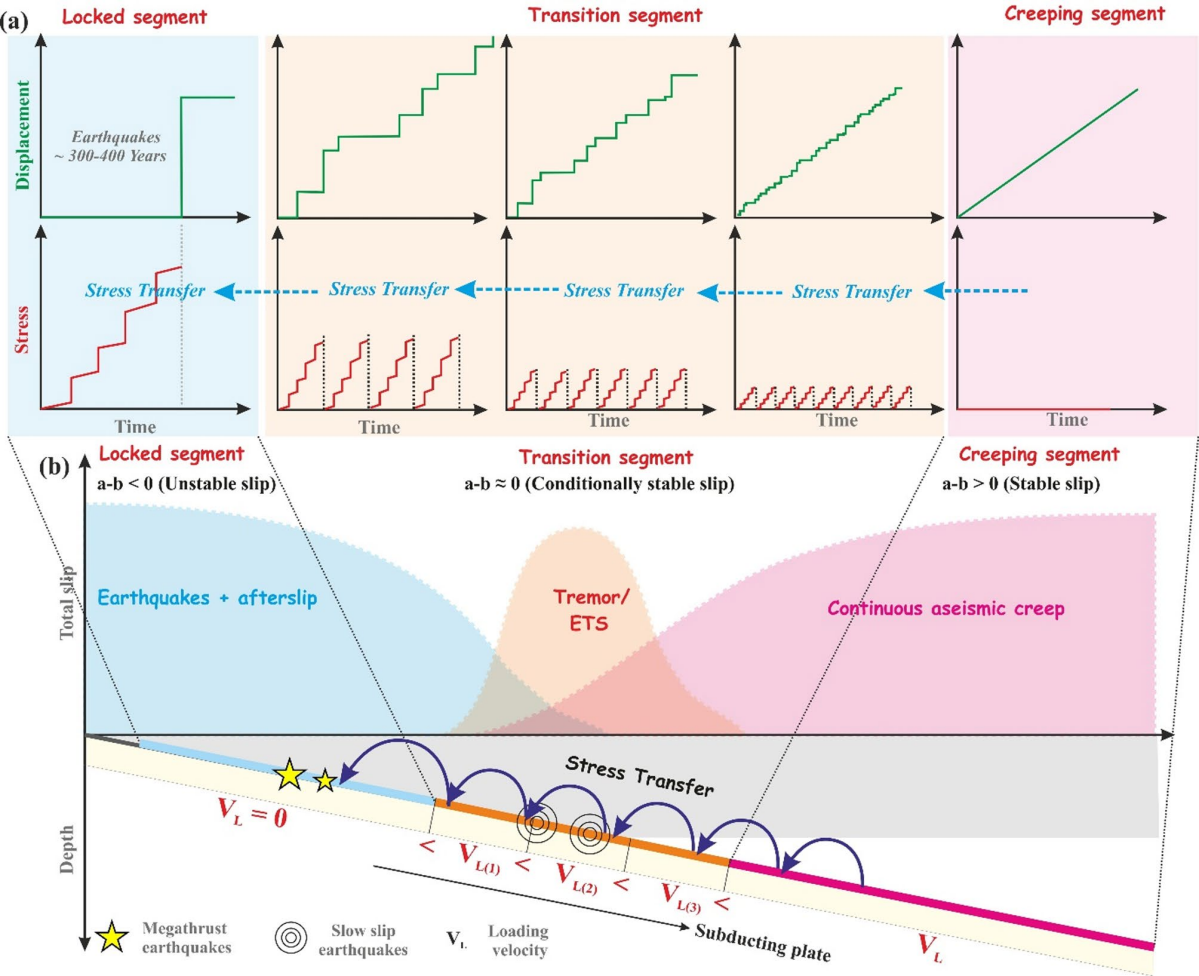
(**a**) Top panel represents the displacement timelines profile from the locked segment to the creeping segments. (Bottom panel) Schematic representation of the stress transfer model. (**b**) Schematic cross-sectional profiles of a subduction zone showing stress transfer from the creeping segment to the locked zone. The creeping segment is weakly coupled and slips easily. Each slip relieves stress locally and transfers stress to a stronger portion of the fault with a higher stress threshold which eventually modulates the timing of the megathrust earthquakes in the locked zone (yellow star).

Next, we investigate variations in seismic activity in terms of quantifiable parameters, like stress drop and slip displacement, with respect to the recurrence time within three segments divided perpendicular to the dip of the slab. These are the locked segment (in blue) where megathrust earthquakes occur as consequence of velocity weakening friction, followed by the transition segment (in yellow) of SST occurrence and a further downdip region of aseismic creep (in pink) that undergoes stable sliding because of velocity strengthening (Fig. 14). Seismic/tremor activity transitions from larger, less frequent in the locked zone to smaller, more frequent in the transition zone (Fig. 14a). It has been proposed that tremor activity initiates down-dip in the creeping zone and subsequently propagates up-dip towards the locked zone^21^. The creeping segment undergoes steady stable slip, which implies constant displacement with respect to time, resulting in loading of the downdip edge of the tremor region^21^. In this model, the relative frictional strength increases as we move up-dip, which implies that subsequent portions towards the trench have a higher stress threshold. Several smaller slow slip episodes are required to reach the slip threshold of the deeper region. Therefore, the number of slip events for a particular time interval, otherwise known as the periodicity, decreases as we move up-dip. A fractal stress transfer process is envisioned here as a down-dip portion transfers a discrete amount of stress via slow slip to the slightly stronger portion above. This process continues until the megathrust region receives enough stress to get destabilized, resulting in a large earthquake.

In Fig. 14b we present an overview of this stress transfer mechanism (modified after Wech and Creager^29^). As we go downdip from the trench, the temperature increases, causing the pore pressure to increase. This reduces the effective normal stress thereby requiring a lesser stress threshold i.e., a weaker fault that fails frequently to accommodate smaller slip per event. From our results (Figs. 10 and 12c), we observed that the recurrence time is inversely proportional to the loading velocity. Here, we propose a velocity transition with respect to the depth of slab subduction that can explain the behaviour of tremor activity and megathrust earthquakes. The aseismic zone creeps at the rate of plate motion, but this velocity decreases as we move updip through the transition zone and theoretically becomes zero at the locked zone. The logarithmic dependency between recurrence time and plate velocity (Fig. 12c–d) can indicate stress accumulation modulated by kinematic coupling. Updip locked zones accumulate stress at rates proportional to V_L_, while downdip creeping zones release stress episodically via slow slip. Stress transfer from SSEs to the seismogenic zone (Fig. 14) is governed by fault stiffness (K) and healing effects (Im et al., 2017), implicitly captured in our simulations through K < Kc​ (critical stiffness). However, our model assumes homogeneous RSF parameters (a, b, and L), whereas natural faults exhibit spatial heterogeneity in rock rheology and pore pressure^61,62^. Future work integrating thermo-hydro-mechanical coupling will refine these relationships. Since we have also observed that the force (or stress drop) is also inversely proportional (Fig. 10a) to the loading velocity, tracking the velocity of deformation through geodetic measurements and seismic monitoring over the transition zone, can act as crucial evidence for stress build-up in the locked zone.

### Concluding discussions: interaction between slow-slip events and megathrust earthquakes

Slow-slip events frequently occur in a brittle-to-ductile transition zone just below the brittle zone where large earthquakes nucleate. It has been argued that slow-slip events may influence the occurrence of megathrust earthquakes and their time of occurrence^6,63–66^. The slow-slip events may interact with the megathrust earthquakes in three possible ways: first, the slow-slip event and megathrust earthquakes have occurred simultaneously^63^. For example, the Papanoa earthquake in 2014 in the Guerrero region of Mexico occurred during an ongoing slow-slip event. Hence, it is believed that the seismic event was triggered by the slow slip events^67^. However, Obara and Kato^6^ suggested that it may be a by-chance occurrence, as we have not observed many such synchronous behaviours. Second, the recurrence interval of the long-term and short-term slow slip events becomes shorter during the interseismic period^60^. Matsuzawa et al.^68^ also suggested that slow slip events and tremors may be able to tell us how stress builds up at the base of the locked area. Finally, slow slip events may stop after a large earthquake and may occur again as they did previously^6^. As both of these results are not validated by natural observations, we are unsure whether this numerical result is applicable to natural systems or a consequence of the simplifying assumptions of the model. Dascher-Cousineau and Bürgmann^69^ investigated the relationship between SSEs and seismicity, finding a statistically significant increase in earthquake rates during SSEs, with a global average rise of 1.24 times the background level. The study reveals that earthquake density is increased within the seismogenic zones of SSEs, demonstrating a spatial correlation. It also identifies regional variations in seismic productivity, with Costa Rica and Mexico showing greater activity compared to regions like Cascadia and Nankai. However, the authors conclude that SSEs have a limited role in the nucleation of major earthquakes, as only a small fraction are associated with significant seismic events.

While early studies established that plate convergence rates and subducting plate age influence maximum earthquake magnitudes^70,71^ recent megathrust events in regions like Sumatra and Tōhoku—where rapid convergence and old plates coexist—highlight limitations in purely kinematic models. Our findings extend these frameworks by incorporating slow earthquake dynamics as a mediator of stress transfer. Specifically, the logarithmic inverse relationship between recurrence intervals (T_r_) and plate convergence rates (V_L_) (Fig. 12c) suggests that slow slip events (SSEs) modulate stress accumulation on megathrusts independently of subducting plate age. This reconciles outliers like Tōhoku, where SSEs near the seismogenic zone^6^ may counteract compressive stress fields predicted by earlier models. Our integrated framework interpretes slow earthquakes as stress-meters by linking their periodicity to plate motion—a step toward predictive models. For example, in Cascadia, frequent SSEs (Figs. 2 and 3) may limit stress buildup despite rapid convergence, whereas in Tōhoku, SSEs near the locked zone^72^ could modulate rupture timing. Critically, our logarithmic scaling (Fig. 12c–e) reconciles disparate findings on SSE-megathrust interactions. For instance, while Audet & Bürgmann^21^ attributed SSE periodicity to silica enrichment, and Dal Zilio et al.^59^ emphasized thermal controls, our results demonstrate that T_r_∝−(V_L_) persists independently of lithospheric heterogeneity. This universality suggests plate kinematics imposes a first-order control, refining the ‘stress-meter’ hypothesis^6^.

However, the exact stress transfer mechanism from ETS segments to up-dip seismogenic zone with the triggering potential of a megathrust earthquake remains equivocal, although it has been explored in a few studies^21,73,74^. A major connection between SSEs and earthquake hazards is not obvious from the observed range of behaviours. Using integrated constraints from natural observations, numerical simulations from the rate and state friction model, and comparisons with laboratory-based experimental observations, it can be suggested that slow slip events can be used as possible stress-meters or a direct proxy of large megathrust earthquakes and probably modulate the megathrust earthquakes in the seismogenic zone.

While prior studies have examined slow slip events (SSEs) and megathrust earthquakes through isolated lenses (e.g., geodetic observations: Bürgmann^18^; Jolivet and Frank^9^; laboratory experiments: Im et al.^25^), our tripartite approach from natural observations of subduction zones along the Pacific Ring of Fire, quasidynamic numerical simulations under the framework of the RSF model, and laboratory-based controlled stick-slip experiments bridges these scales. The results can be summarised by the following key points: (1) the recurrence interval of great earthquake cycle and SSTs depends upon long-term fault motion or plate velocity. We report that the recurrence interval of the earthquake cycle decreases logarithmically with increasing loading velocity. (2) the logarithmic dependency between recurrence time and loading velocity of the stick-slip motion in simple frictional models and laboratory experiments also appears to complement the depth dependency of the tremor activities and associated slip-periodicity observed in the Cascadia and the Nankai subduction zones. The slip distribution patterns of these subduction zones match with the model-predicted displacement for corresponding loading velocity, which never exceeds the down-dip plate motion along these subduction zones. (3) we suggest that the slow slip events occurring in the deeper depth (i.e., brittle-to-ductile transition zone) slip more frequently due to their higher localised slip accumulation rate and may episodically transfer stress to the locked zone which remains locked or is stressed by lower long term plate motion. Therefore, slow earthquakes can serve as stress-meters for megathrust earthquakes.

### Limitations and future works

Further, slow earthquakes, more frequent than megathrust events, provide critical insights into rupture mechanics and recurrence cycles, often missing from megathrust records. The assumption that complementary distributions of ETS/slow earthquakes and megathrust fast earthquakes exist, although conjectural, helps identify the landward extent of potential megathrust events, vital for seismic hazard assessment. However, challenges like limited access to deep subduction zones and integrating deformation across vast timescales persist for megathrust earthquake cycles. Laboratory stick-slip experiments and numerical simulations address these gaps by replicating seismic behaviours, refining fault mechanics models and seismic hazard assessment studies.

Our quasi-dynamic model employs rate-and-state friction (RSF) with radiation damping, assuming constant effective normal stress, homogeneous fault properties, and simplified thermal/pressure gradients. While this framework captures first-order depth-dependent slip periodicity, it omits critical processes influencing natural subduction zones. For instance, fluid-driven mechanisms such as permeability evolution and episodic pressurization^20^ are excluded, yet these modulate slip behavior and stress transfer efficiency. Elevated pore pressures in fluid-rich margins like Nankai may enhance slow-slip event (SSE)-megathrust interactions^60^ whereas subduction zones like Chile having lower permeability likely exhibit weaker coupling—factors contributing to scatter in recurrence intervals (Fig. 12b). Furthermore, our simulations assume spatially uniform RSF parameters (*a*, *b*, and *L*), neglecting natural heterogeneity in rock rheology and lithology^61,62^. Small-scale heterogeneities and fully dynamic rupture propagation also remain unexplored. Future studies should integrate thermo-hydro-mechanical (THM) coupling to resolve depth-varying frictional stability and pore-pressure dynamics. Incorporating fluid-driven processes (e.g., dilatancy, permeability feedbacks) thermal regimes and heterogeneous fault geometries would refine linkages between SSEs and megathrust earthquakes, bridging laboratory-scale parameters to field-scale conditions, strengthening the “stress-meter” hypothesis and enhance seismic hazard forecasts.

## Materials and methods

### Numerical modelling approach

To simulate the recurrence time of the earthquake cycles, we consider a single-degree-of-freedom spring block system, where a block is pulled on a frictional base through a spring at a constant loading velocity V_L_ (Fig. S1). The equilibrium of forces is acting on the block that can be expressed quasi-dynamically with the following equation of motion^75^ by considering the spring constant K, the loading velocity V_L_, and the frictional shear stress (τ). The rate of change of the frictional shear force at the interface of the sliding surface by neglecting the inertia of the system (i.e., quasi-static condition) is given by:

3 $$\:\frac{d\tau\:}{dt}=K\left({V}_{L}-V\right)$$

Assuming the frictional stress at the base of the block obeys the rate-and-state-dependent friction law, in which the shear force (τ) depends upon the slip velocity (V) and state of the sliding surface ($\:\theta\:$)^76^. Based on the experimental studies of rock friction, the empirical rate and state friction law^75,77^ is expressed as:

4 $$\:\tau\:={\sigma\:}_{n}\left[{\mu\:}_{0}+aln\left(\frac{V}{{V}_{*}}\right)+bln\left(\frac{{V}_{*}\theta\:}{L}\right)\right]$$

where $\:{\mu\:}_{0}$ is the frictional coefficient, $\:{\sigma\:}_{n}$ is the effective normal stress on the block’s sliding surface, $\:{V}_{*}$ is the arbitrary reference velocity, $\:\theta\:$ is the state of the sliding surface^76^ L is the critical slip distance and a, b are the frictional parameters. Further, the recurrence time of slow earthquakes in the deeper segments of the seismogenic extension of a usual earthquake fault is modelled as a function of changes in the load-point velocity (i.e., pulling velocity), such that the impact of the repeating slow earthquakes can be integrated into subsequent loading history.

To analyze the above equations in their dimensionless form, we introduce the following non-dimensional variables:$\:\:ln\left(\frac{V}{{V}_{*}}\right)=\varnothing\:$,$\:\:ln\left(\frac{{V}_{*}\theta\:}{L}\right)=\widehat{\theta\:}$, $\:\frac{\tau\:}{{\sigma\:}_{n}a}=\phi\:$, $\:T=\frac{t{V}_{*}}{\:L}$, $\:\beta\:=\frac{\:b}{a}$, $\:{v}_{0}=\frac{{V}_{L}\:}{{V}_{*}}$, $\:\frac{{\mu\:}_{0}}{{a}_{1}}={\mu\:}_{*}$

The non-dimensional form of the Eq. (2) is given by:

5 $$\:\phi\:={\mu\:}_{*}+\varnothing\:+\beta\:\widehat{\theta\:}$$

6 $$\:\widehat{\theta\:}\:=\frac{\phi\:-\:\varnothing\:-{\mu\:}_{*}}{\beta\:}$$

By taking the derivative of the Eq. (3) and expressed as:

7 $$\:\frac{d\phi\:}{dT}=\frac{d\varnothing\:}{dT}+\beta\:\frac{d\widehat{\theta\:}}{dT}$$

Here, we have considered the ageing law^23^ to solve the single spring-block RSF model, which is expressed as:

8 $$\:\frac{d\theta\:}{dt}\:=1-\frac{V\theta\:}{L}$$

The non-dimensional form of the Eq. (6) can be expressed as:

9 $$\:\frac{d\theta\:}{dt}=1-{e}^{\varnothing\:}{e}^{\widehat{\theta\:}}$$

As we consider $\:T=\frac{t{V}_{*}}{L}$

10 $$\:\frac{dT}{dt}=\frac{{V}_{*}}{L}\:\:\:$$

Similarly

$$\:\widehat{\theta\:}=\frac{{V}_{*}\theta\:}{L}$$

11 $$\:\frac{d\widehat{\theta\:}}{dT}=\frac{d\theta\:}{dT}\:\frac{1}{\theta\:}=\frac{d\theta\:}{dt}\:\frac{dt}{dT}\:\frac{1}{\theta\:}$$

By replacing Eq. (7) and Eq. (8) in Eq. (9)

12 $$\:\frac{d\widehat{\theta\:}}{dT}=\left({e}^{-\widehat{\theta\:}}-{e}^{\varnothing\:}\:\:\right)$$

Similarly

$$\:\frac{\tau\:}{{\sigma\:}_{n}a}=\phi\:$$

13 $$\:\frac{d\phi\:}{dT}=\frac{1}{A}\frac{d\tau\:}{dT}=\frac{1}{A}\frac{d\tau\:}{dt}\:\frac{dt}{dT}$$

Now replaceing $\:\frac{d\tau\:}{dt}$ and $\:\frac{dt}{dT}$ value in the Eq. (11)

$$\:\frac{d\phi\:}{dT}=K\left({V}_{0}-V\right)\frac{L}{A{V}_{*}}$$

14 $$\:\frac{d\phi\:}{dT}={k}_{1}\left({v}_{0}-{e}^{\varnothing\:}\right)\:\text{w}\text{h}\text{e}\text{r}\text{e}\:{k}_{1}=\frac{KL}{A}$$

By combining Eqs. (5) and (12), the non-dimensional equation for a slip can be expressed as:

15 $$\:\frac{d\varnothing\:}{dT}=\:{k}_{1}\left({v}_{0}-{e}^{\varnothing\:}\right)\:\:-\beta\:\left({e}^{-\left(\frac{\phi\:-\:\varnothing\:-{\mu\:}_{*}}{\beta\:}\right)}-{e}^{\varnothing\:}\:\right)$$

Finally, the two non-dimensional equations for the spring-block model can be expressed as:

16 $$\:\frac{d\varnothing\:}{dT}=\:{k}_{1}\left({v}_{0}-{e}^{\varnothing\:}\right)\:\:-\beta\:\left({e}^{-\left(\frac{\phi\:-\:\varnothing\:-{\mu\:}_{*}}{\beta\:}\right)}-{e}^{\varnothing\:}\:\right)$$

17 $$\:\frac{d\phi\:}{dT}={k}_{1}\left({v}_{0}-{e}^{\varnothing\:}\right)$$

Equations (1, 2 and 6) are solved numerically using ordinary differential equation solver ode45 under the MATLAB platform.

### Laboratory experimental details on stick-slip instability

The laboratory-based experiments were conducted at constant room temperature (~ 25 °C) conditions, considering a single-degree freedom spring-block model, which is the simplest model that reproduces the earthquake cycle, including megathrusts and slow earthquakes (Fig.S2 and Table T1). Detailed laboratory-based experimental setup along with individual comments complementing stick-slip frictional instability has been presented in Figure S2. The rock sample (5.0 × 5.0 × 1.5 cm^3^) placed on the frictional sliding surface is attached to a force sensor that is connected to the linear slider by a sample holder. Further, the linear slider is attached to the computer-controlled servo motor system, which pushes or pulls the sample with a constant velocity over the frictional surface. When the sample is pushed or pulled with a constant velocity, the shear force develops at the frictional interface of the rock sample and the rough surface. This shear force is measured using the digital force sensor connected to a data acquisition system which records the signal in terms of voltages and the accelerometer on top of the sample records the instantaneous acceleration. This recorded voltage is converted to force with ± 0.1 N precision. Before converting voltage to force, the load cell is calibrated by increasing the load and calculating the output signal at each step. From the load versus voltage graph, the load is estimated by multiplying the slope of the load versus voltage graph with the voltage value. The surface roughness of both the sample (or sliding block) and the frictional surface has been also quantified using the surface roughness tester (Fig. S3), which is characterized in terms of mean roughness (R_z_). The mean roughness (R_z_) is defined as the average value of the heights of the five highest-profile peaks and the depths of the five deepest valleys (Fig. 8 and Fig. S4) and mathematically it can be expressed as:

18 $$\:{R}_{z}=\:\frac{1}{5}{\sum\:}_{j=1}^{5}\left({Z}_{{P}_{j}}+{Z}_{{V}_{j}}\right)$$

where, $\:{R}_{z}$ is the average maximum height of the profile,$\:\:{Z}_{{P}_{j}}$ is the height of peaks and $\:{Z}_{{V}_{j}}$ is the depth of valleys. The higher value of the mean roughness (R_z_) indicates more irregularity of the surface and vice-versa. Further, the scanning electron microscopy (SEM) images of the sample and the frictional surface under three different magnifications also indicate that the rock sample surface is smoother than the frictional surface.

## Electronic supplementary material

Below is the link to the electronic supplementary material.

Supplementary Material 1

## Data Availability

We used the tectonic tremor catalogue of the Nankai subduction zone, western Japan, and the Cascadia subduction zone, in North America, which can be archived from the World Tremor database (http://www-solid.eps.s.u-tokyo.ac.jp/~idehara/wtd0/Welcome.html). Here, we have analyzed the tectonic tremor catalog to infer the location and duration of the slow slip in the Cascadia subduction and Nankai subduction zone from 2005 to 2013 and 2001-2016, respectively. The recurrence time of megathrust earthquakes (of > M7.0-M9.6 hosted within 32 trench segments) and slow earthquakes and the range of convergence rates across various subduction segments presented in this article are obtained from the previous literatures mentioned in the supporting document. Nevertheless, we have also presented these compiled datasets in the supplementary tables T2 and T3 of the supporting document.
