# Supplementary material for: Role of plate convergence rate in shaping earthquake recurrence in subduction zones
Source: Sci Rep. 2025 Jul 1;15:21227. doi: 10.1038/s41598-025-04766-y (PMC12214789; doi:10.1038/s41598-025-04766-y)
Supplement: Supplementary file 1 — Supplementary Material 1 [file 41598_2025_4766_MOESM1_ESM.docx]

**Supporting document for**

**Role of Plate Convergence Rate in Shaping Earthquake Recurrence in Subduction Zones**

**Sayak Ray^1^, Bhaskar Kundu^1*^, Batakrushna Senapati^2^, Abhijit Ghosh^3^, Arun K. Singh^4^**

^1^Department of Earth and Atmospheric Sciences, NIT Rourkela, Rourkela, 769008, India

^2^Department of Earth Science, National Central University, No. 300, Jhongda Rd., Chungli, Taoyuan 320, Taiwan

^3^Department of Earth and Planetary Sciences, University of California Riverside, USA

^4^Department of Mechanical Engineering, VNIT, Nagpur, Maharashtra, 440010, India

***Corresponding author:** Bhaskar Kundu, Department of Earth and Atmospheric Sciences, NIT Rourkela, Rourkela, 769008, India, (email: [rilbhaskar@gmail.com](mailto:rilbhaskar@gmail.com)).

**This supplementary document contents:**

**Additional information text**

**Supplementary Figures S1 – S11.**

**Supplementary Tables T1 – T4.**

**Physical constraints of Rate and state-dependent friction model**

The Rate and State Friction (RSF) model, which describes how frictional strength evolves with sliding rate and time, operates within specific physical constraints. These include factors such as inertia (or mass), damping coefficient, temperature, and subduction zone fault dip, all of which can significantly influence the model's outcomes.

*(a) Role of inertia/mass*

To explore the effect of the mass/inertia on the rate and state-dependent friction model, we have numerically evaluated the rate and state-dependent friction model considering a block having mass “m” that slides frictionally with velocity V, applying a constant velocity V_L_ imposed at the other end of the spring. The rate of change of the frictional shear force (τ) developed at the base of the block can be expressed as:

$\frac{d\tau}{dt}=K\left( V_{L}-V \right)-m\frac{d^{2}V}{dt^{2}}$ (19)

Here, we have solved equation (2), equation (6), and equation (19) in non-dimensional form by introducing the following non-dimensional variables:$ln\left( \frac{V}{V_{*}} \right)=\emptyset$,$\left( \frac{V_{*}\theta}{L} \right)=\hat{\theta}$ , $\frac{\tau}{\sigma_{n}a}=\varphi$ , $T=\frac{tV_{*}}{L}$ , $\beta=\frac{b}{a}$ , $v_{0}=\frac{V_{L}}{V_{*}}$ , $\frac{\mu_{0}}{a_{1}}=\mu_{*}$ following Ranjith and Rice^79^ approach.

Finally, the two non-dimensional equations for the rate and state-dependent friction model are expressed as:

$\left. \begin{aligned} \frac{d\varphi}{dT}=\frac{d\emptyset}{dT}+\beta\left( e^{-\left( \frac{\varphi- \emptyset-\mu_{*}}{\beta} \right)}-e^{\emptyset} \right) \\ \frac{d^{2}\emptyset}{dT^{2}}=\frac{k_{1}\left( v_{0}-e^{\emptyset} \right)-\frac{d\varphi}{dT}}{re^{\emptyset}}-\left( \frac{d\emptyset}{dT} \right)^{2} \end{aligned} \right\}$ (20)

We solved equation (20) numerically using MATLAB’s ordinary differential equation solver ode45 (for details, see supporting document). From this numerical solution, we observed that the mass of the block increases the amplitude of slip velocity and shear stress of the system. However, it does not affect the dynamic stability of the system (Fig. S5). Im et al.^80^ also observed that the mass of the block has very little effect on the rate and state-dependent friction model. Therefore, we argue that the effect of inertia/mass of the block in the numerical model simulation (and in the dependent analysis of the results section) will not cause any substantial change in the outcome.

*(b) Role of damping coefficient*

When we introduced the damping coefficient in the rate and state-dependent friction model, the rate of change of the frictional shear force (τ) developed at the base of the block can be expressed as:

$\frac{d\tau}{dt}=K\left( V_{L}-V \right)+\frac{GV}{2V_{s}}$ (21)

where $\frac{GV}{2V_{s}}$ is the damping coefficient, $V_{s}$ is the S-wave velocity, and G is the rigidity modulus. Here, we have also solved equation (2), equation (6), and equation (21) in non-dimensional form by introducing the following non-dimensional variables:$ln\left( \frac{V}{V_{*}} \right)=\emptyset$,$\left( \frac{V_{*}\theta}{L} \right)=\hat{\theta}$ , $\frac{\tau}{\sigma_{n}a}=\varphi$ , $T=\frac{tV_{*}}{L}$ , $\beta=\frac{b}{a}$ , $v_{0}=\frac{V_{L}}{V_{*}}$ , $\frac{\mu_{0}}{a_{1}}=\mu_{*}$,$c=\frac{GL}{2AV_{s}}$ following Ranjith and Rice^79^ approach. Finally, the non-dimensional equation for rate and state-dependent friction model considering damping coefficient are expressed as:

$\left. \begin{aligned} \frac{d\varphi}{dT}=k_{1}\left( v_{0}-e^{\emptyset} \right)+ce^{\emptyset} \\ \frac{d\emptyset}{dT}=k_{1}\left( v_{0}-e^{\emptyset} \right)+ce^{\emptyset}-\beta\left( e^{-\left( \frac{\varphi- \emptyset-\mu_{*}}{\beta} \right)}-e^{\emptyset} \right) \end{aligned} \right\}$ (22)

From the numerical simulation, it has been observed that the damping coefficient only increases the slip velocity and shear stress of the system. It does not affect the dynamic stability of the system (Fig. S6), hence we neglect the damping coefficient from our numerical model simulation and the present study.

*(c) Role of megathrust dip*

To understand the effect of dip or inclination of the subduction zone on the rate and state-dependent friction model, we considered a sliding block that makes an angle (α) with the surface. The sliding block moves with a velocity V when a constant velocity V_L_ is imposed at the other end of the spring (Fig. S7). The rate of change of the frictional shear force (τ) at the interface of the sliding surface by neglecting the mass of the system is expressed as:

$K\left( V_{L}t-x \right)+\sin\alpha=\tau$ (23)

By taking the derivative of the equation (23), it can be expressed as:

$\frac{d\tau}{dt}=K\left( V_{L}-V \right)$ (24)

From equation (24), it is noticed that the rate of shear stress is independent of the slope angle. Hence, the slope of the block does not affect the dynamic stability of the sliding mass. The two non-dimensional equations for rate and state-dependent friction models considering the dip of the block are expressed as:

$\left. \begin{aligned} \begin{aligned} \frac{d\emptyset}{dT}= k_{1}\left( v_{0}-e^{\emptyset} \right) -\beta\left( e^{-\left( \frac{\varphi- \emptyset-\mu_{*}}{\beta} \right)}-e^{\emptyset} \right) \\ \frac{d\varphi}{dT}=k_{1}\left( v_{0}-e^{\emptyset} \right) \end{aligned} \\ \mathrm{where} k_{1}=\frac{KL}{(a.w)cos\alpha} \end{aligned} \right\}$ (25)

From these non-dimensional equations (25) for the rate and state-dependent friction model with the dip angle, it has been observed that the dip of the frictional surface increases only the slip velocity and shear stress of the system. It does not affect the dynamic stability of the system, hence we have neglected such factors.

*(d) Effect of temperature and other physical parameters*

From the numerical study of the rate, state, and temperature friction (RSTF) model, it has been proposed that the increase in the temperature of the sliding interface due to shear heating reduces the strength of the contact asperities^81^. As a result, the earthquake nucleation time of the heated surface is slightly earlier than the unheated surface. Lorenzo and Loddo^82^ studied the earthquake process by considering extended Kato’s RSTF model approach and suggested that the pore pressure can accelerate the nucleation process due to shear heating. Beeler et al.^83^ performed step velocity as well as step temperature experiments and observed that sliding velocity and temperature of sliding surfaces have the opposite effect on steady friction. Singh and Singh^84^ studied the stability of the RSTF model and proposed that the critical stiffness of the heated surface may increase or decrease from the corresponding critical stiffness of the unheated surface. Wang & Barbot^85^ argued that the coupling between shear heating and the temperature dependence of frictional resistance and contact healing might be responsible for the development of slow-slip events in the velocity-strengthening domain below the seismogenic zone. Therefore, it is important to understand the effect of the temperature on the rate and state-dependent friction model, as it varies for different subduction zones^86^.

When the block slides on the surface, it generates shear heating at the slip interface. This heat energy is transferred to the surrounding rock media and vice-versa. The equation of energy balance^87,88^ can be expressed as:

$\rho_{s}c_{v}\frac{dT_{s}}{dt}=\tau\frac{v}{h}-\frac{2q_{h}}{h}$ (26)

When the thickness of the shear zone (h) is negligible, the equation (26) can be expressed as:

$\frac{dT_{s}}{dt}=\frac{\tau v}{{2\rho}_{s}c_{v}h_{w}}-\frac{K_{T}}{{\rho_{s}c_{v}h_{w}h}_{w}}\left( T_{s}-T_{a} \right)$ (27)

where $\rho_{s}$ is the density of the block, $c_{v}$ is specific heat energy, $q_{h}$ is the heat flux, $h_{w}$ is the thickness of the damage zone, $K_{T}$ thermal conductivity,$\frac{v}{h}$ shear strain rate, h is the thickness of the shear zone, $T_{s}$ the temperature of the sliding of the surface and $T_{a}$ is the temperature of the surroundings of the sliding interface.

Following the Chester and Higgs^89^ and Chester^90^ experimental observations, the rate, state and temperature-dependent friction (RSFT) model can be expressed as:

$\tau=\sigma_{n}\left( \mu_{0}+aln\left( \frac{V}{V_{*}} \right)+bln\left( \frac{V_{*}\theta}{D_{c}} \right) \right)+\frac{Q_{a}a-Q_{b}b}{R}\left[ \frac{1}{T_{s}}-\frac{1}{T_{*}} \right]$ (28)

where $\mu_{0}$ coefficient of friction, $T_{*}$ reference temperature, $T_{s}$ the temperature of the sliding surface, Q_a_ and Q_b_ activation energy corresponding to frictional constant a and b.

To analyze the above equations in their dimensionless form, we introduce the following variables:$ln\left( \frac{V}{V_{*}} \right)=\emptyset$ , $\left( \frac{V_{*}\theta}{D_{c}} \right)=\hat{\theta}$ , $\frac{\tau}{\sigma_{n}a}=\varphi$ , $T=\frac{tV_{*}}{D_{c}}$ , $\beta=\frac{B}{A}$ and $v_{0}=\frac{V_{L}}{V_{*}}$ , $\hat{T_{s}}=\frac{T_{s}}{T_{*}}$, $\frac{\sigma_{n}\left( Q_{a}-Q_{b}\beta\right)}{RT_{*}}=G$, $\frac{\mu_{0}}{a}=\mu_{*}$

Finally, the non-dimensional equation for the RSFT model can be expressed as:

$\left. \begin{aligned} \begin{aligned} \frac{dT_{s}}{dt}=a_{1}\varphi e^{\emptyset}-a_{2}\left( \hat{T}_{s}-\hat{T}_{a} \right) \\ \frac{d\emptyset}{dT}=k_{1}\left( v_{0}-e^{\emptyset} \right)+\beta e^{\emptyset}-\beta e^{-\left( \frac{{\varphi-\mu}_{*}-\emptyset-G\left( \hat{T}_{s}^{-1}-1 \right)}{\beta} \right)}+ G\hat{T}_{s}^{-2}\left[ a_{1}\varphi e^{\emptyset}-a_{2}\left( \hat{T}_{s}-\hat{T}_{a} \right) \right] \\ \frac{d\varphi}{dT}=k_{1}\left( v_{0}-e^{\emptyset} \right) \end{aligned} \\ \mathrm{where}a_{1}=\frac{Ad_{c}}{{2\rho}_{s}c_{v}h} ; a_{2}= \frac{K_{T}d_{c}T_{*}}{{V_{*}\rho}_{s}c_{v}hh_{w}} \end{aligned} \right\}$ (29)

From the numerical simulation of equation (29), it has been observed that the temperature does not affect the shear stress of the system, whereas it may influence the slip velocity and stability dynamics of the system (Fig. S8).

The rate and state-dependent friction model also depends upon the critical slip distance (L) and normal stress (σ_n_). To investigate the effect of these physical parameters on the rate and state-dependent friction model, we systematically vary the critical slip distance at constant loading velocity and normal stress (Fig. S9). The recurrence time and amplitude of slip velocity and frictional coefficient slightly increase with increasing the critical slip distance (Fig. S9). Similarly, we have also systematically varied the normal stress at constant loading velocity and critical slip distance and observed that recurrence time and amplitude of slip velocity and frictional coefficient slightly increase with increasing the normal stress (Fig. S10), i.e., higher the normal stress load on the spring–block system, larger the recurrence interval of the stick-slip cycle and vice versa.

**Supporting figures:**


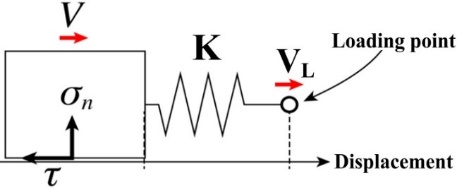


**Figure S1**. Single degree of freedom spring-block model. A block is attached to the spring having stiffness K and pulling with a constant velocity$V_{L}$ against a flat surface. The block generates frictional resistance force τ when the block moves with a velocity of V.


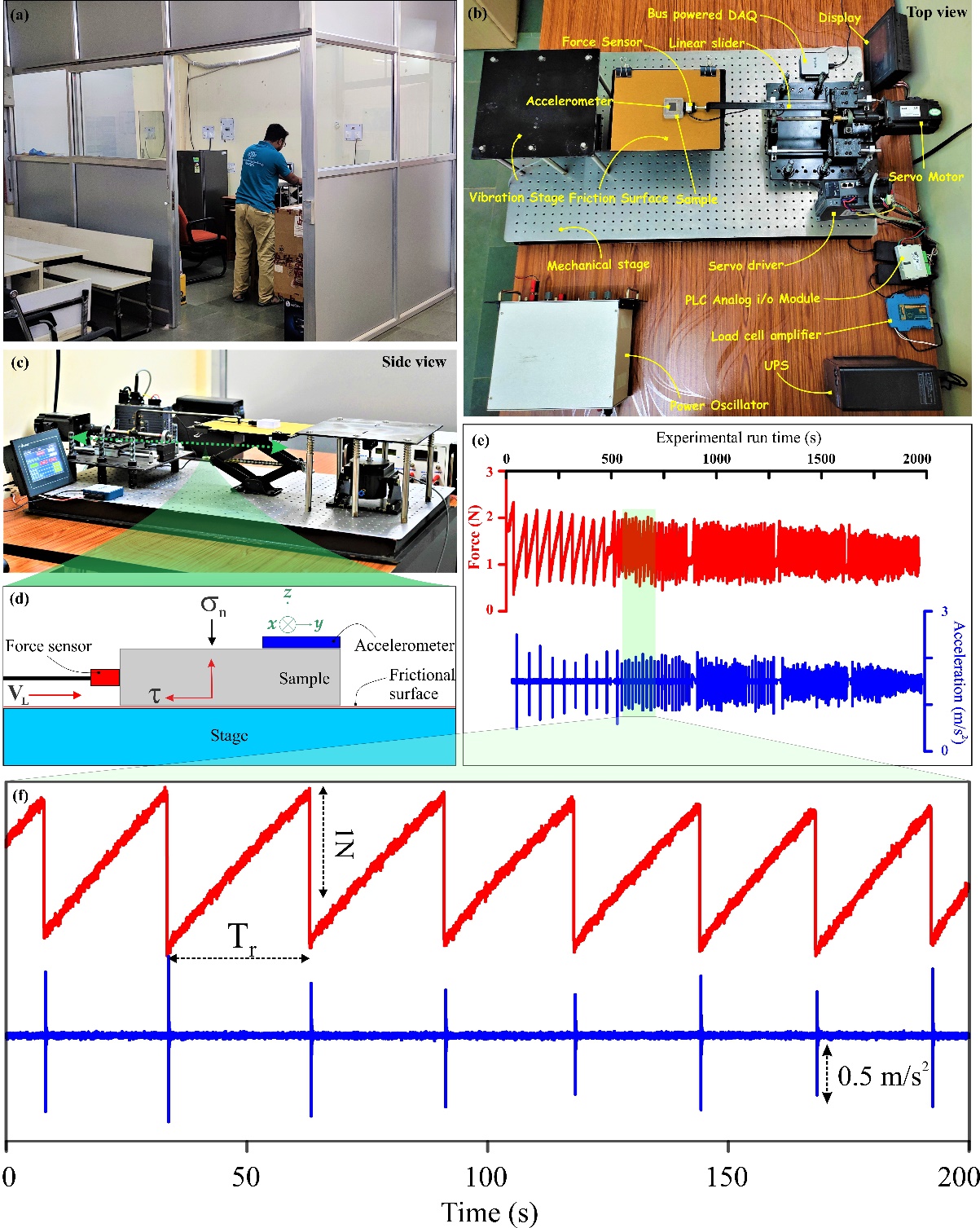


**Figure S2.** (a) Overview of the in house facility of Tectonic Geodesy lab in NIT Rourkela. Top (b) and side (b) view of the frictional laboratory setup with all the components marked in yellow. (d) A schematic diagram of the single degree freedom frictional experiment. The data recorded from the force sensor *(red)* and accelerometer *(blue)* for a single experiment of 1000s runtime is presented in (e). Zoomed in view for 200s of force and acceleration data.


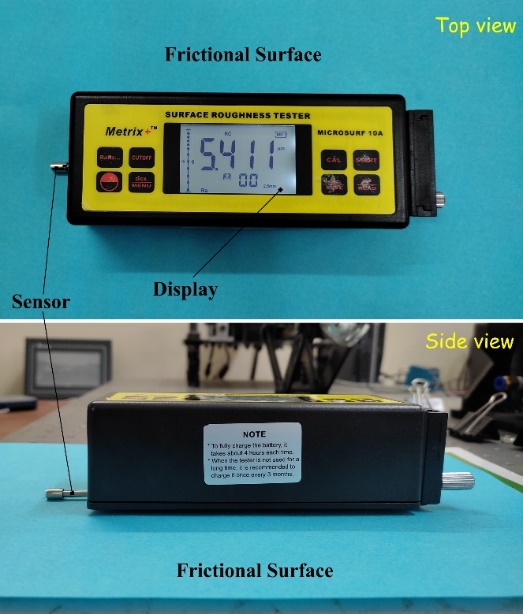


**Figure S3.** Microsurf surface roughness sensor instrument used to measure the various surface roughness parameters


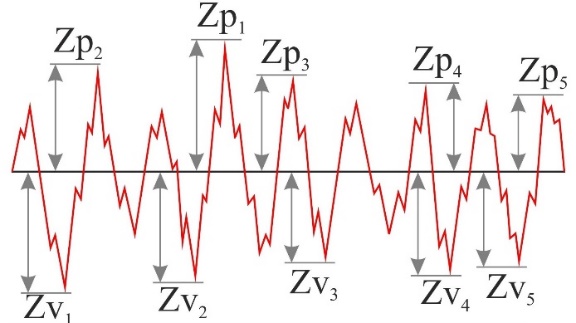


**Figure S4.** Schematic of surface roughness parameter, R_z_ (average maximum height of the profile). Z_p_ is height of the peaks and Z_v_ is depth of the valleys.


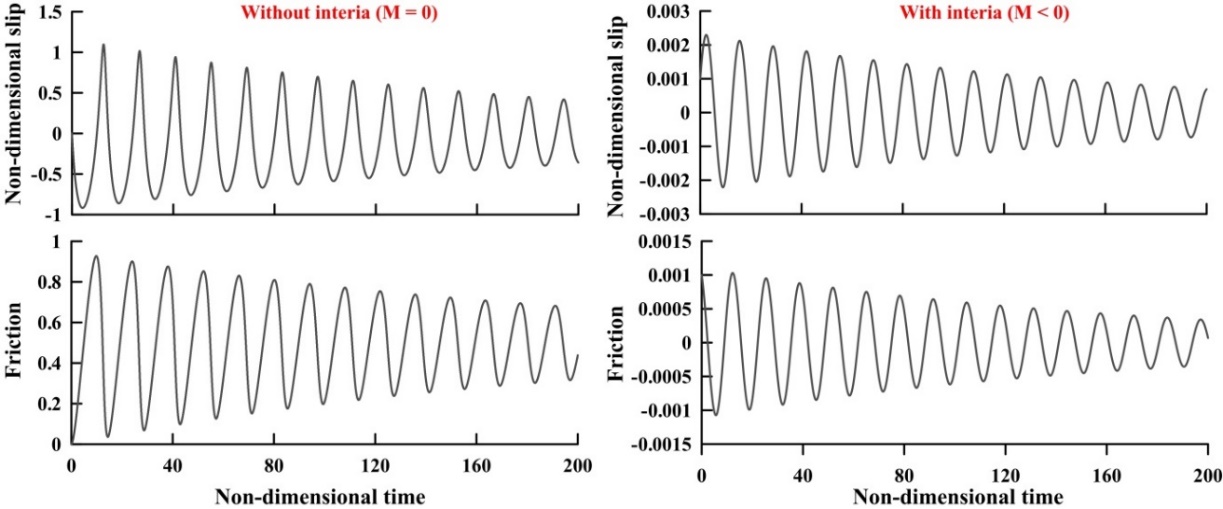


**Figure S5**. Variation of slip and friction as a function of time by considering β=1.2 and K=0.21(left panel) and β=1.2 and K=0.45 (right panel). Note that amplitude of both friction and slip smaller by considering inertia in spring-block model (right panel) as compare to without considering inertia spring-block model.


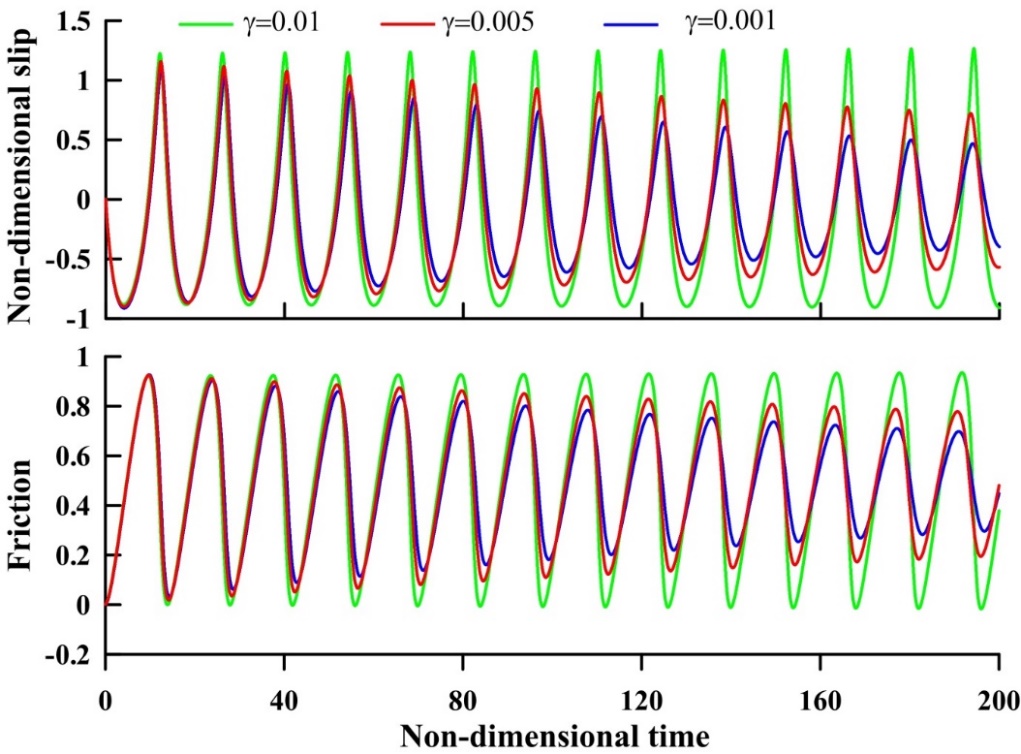


**Figure S6**. Variation of slip and friction as a function of time by considering β=1.2 and Kc=0.21. The green, red and blue curves indicate slip and friction for 0.01, 0.005 and 0.001 damping coefficient respectively. Note that the amplitude of both friction and slip increases with the damping coefficient.


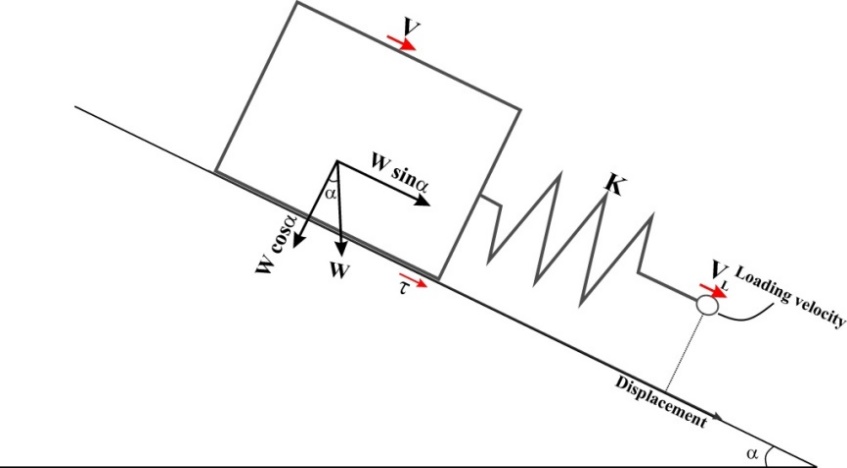


**Figure S7**. Single degree of freedom spring-block model. A block is attached to the spring having stiffness K and making an angle α with the horizontal surface. The block moves with a velocity of V when a constant velocity$V_{L}$ is imposed on the other end of the spring.


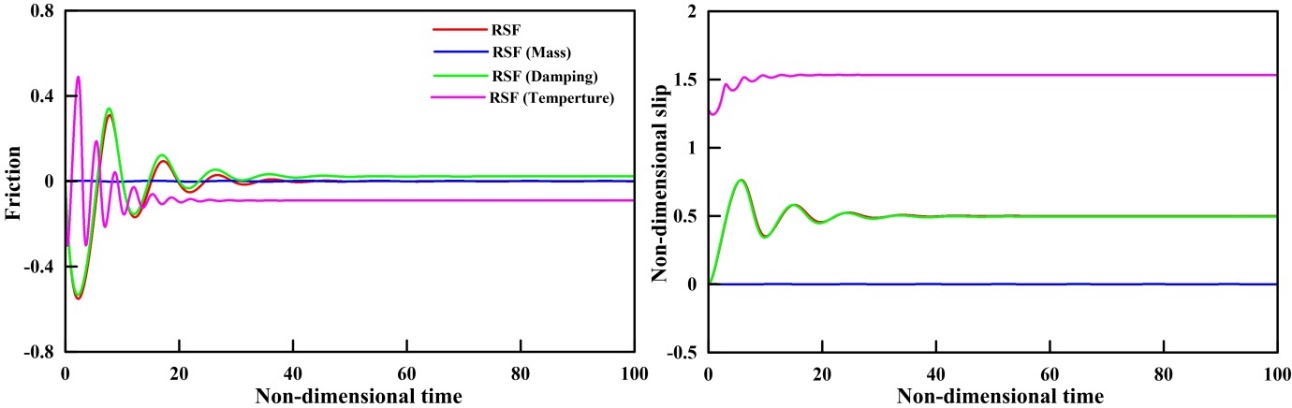


**Figure S8.** Role of mass, damping coefficient, and temperature on friction and slip of the rate and state friction model considering k = 0.45, β = 1.2 and $v_{0}=1$. The Left and right panel represent the variation of friction and slip as a function of time.


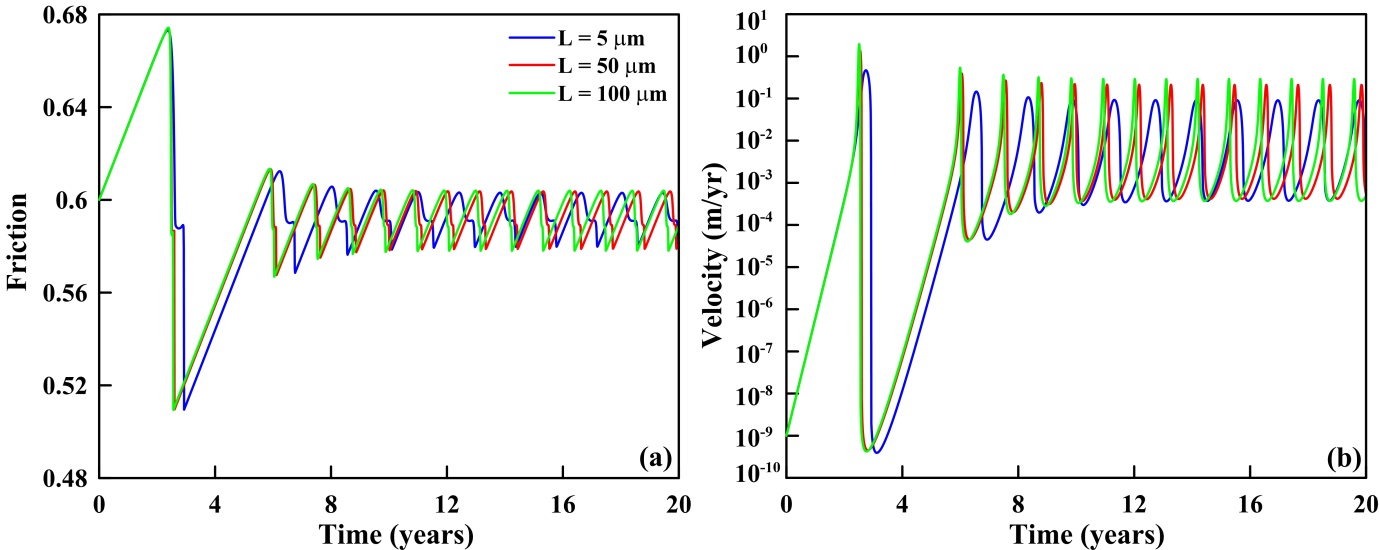


**Figure S9**. The friction and velocity response during the stick-slip motion for various characteristic slip distances (i.e., L= 5, 50 and 100 µm), considering as a= 0.005, b= 0.007, V_L_= 20 mm/yr, σ_n_ = 1 Mpa. Note that the recurrence time slightly increases with increasing of L.


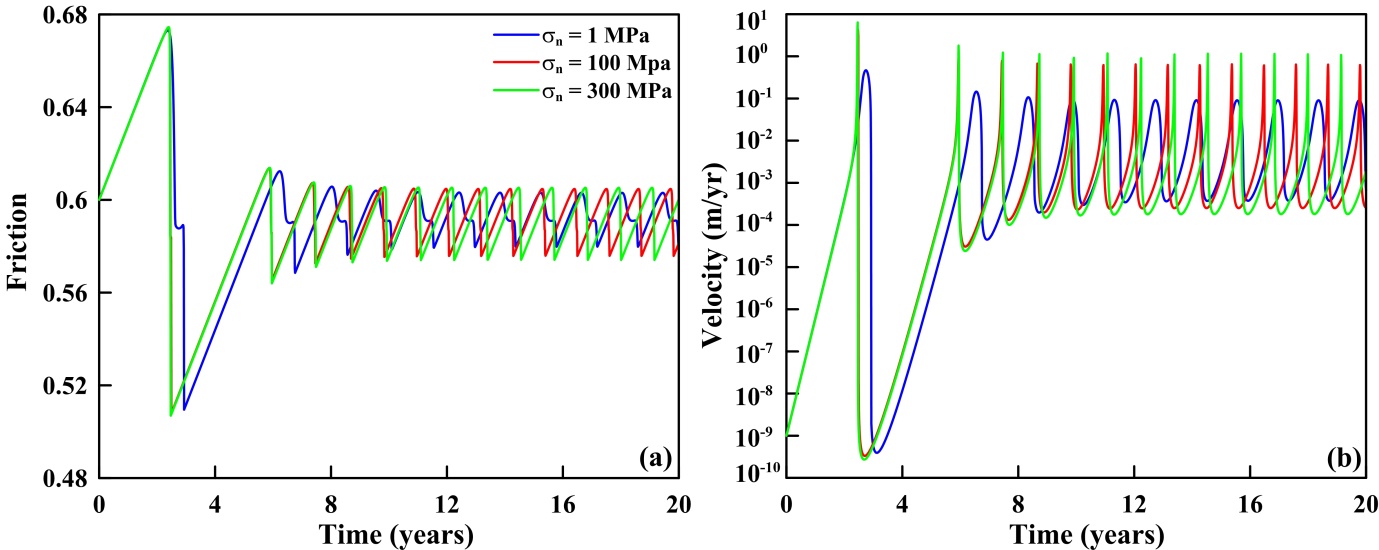


**Figure S10**. The friction and velocity response during the stick-slip motion for different normal stress (i.e., σ_n_ = 1, 100 and 300 MPa), considering as a= 0.005, b= 0.007, V_L_= 20 mm/yr, L= 5 µm. Note that the recurrence time also slightly increases with increasing of σ_n_.

**
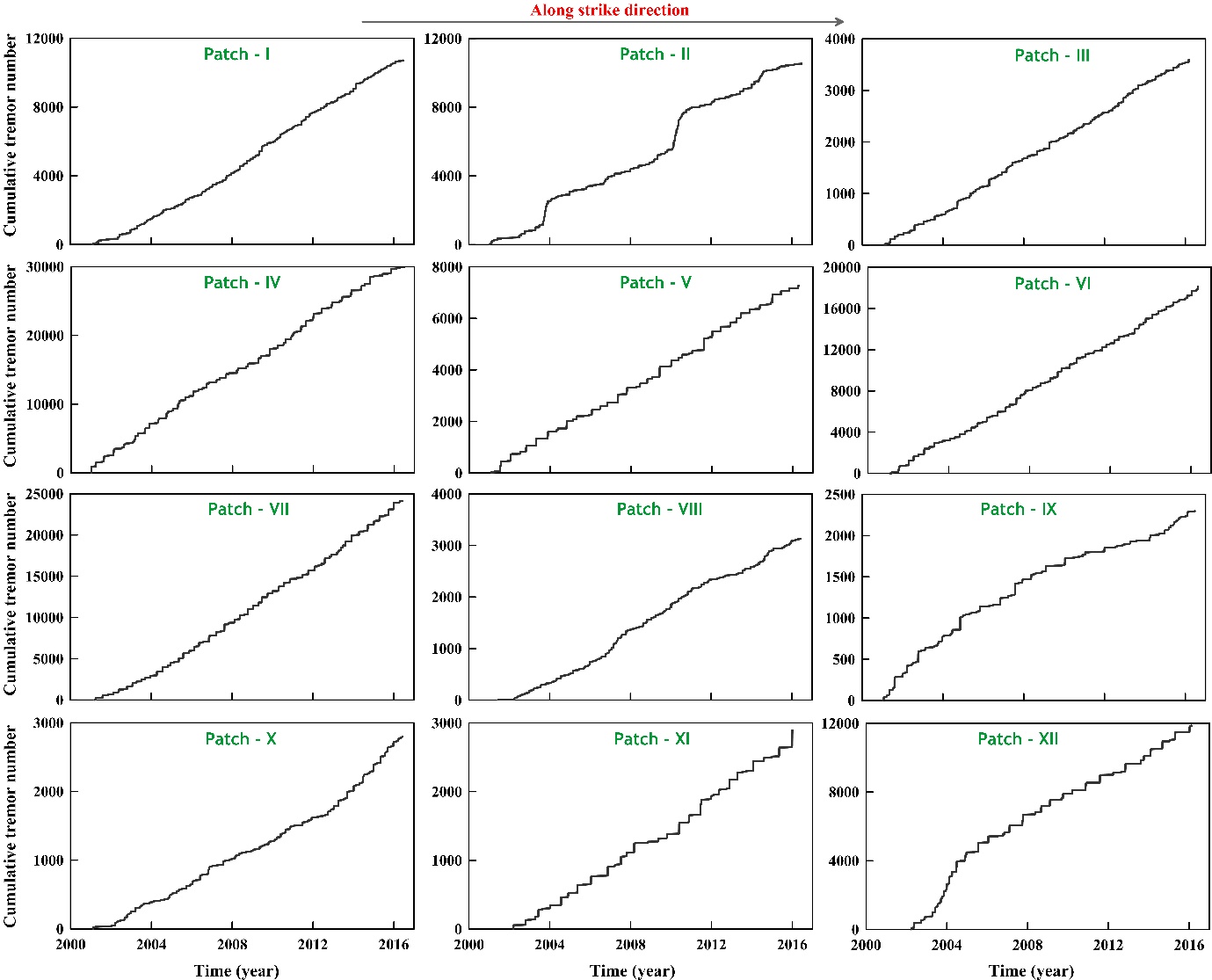
**

**Figure S11.** Variation of cumulative tremor number with time for all tremor patches (shown in Figure 2a). Note that the tremor patches show a transition from the small frequent slip events in the down-dip direction to larger, less frequent slip episodes in the up-dip direction.

**Supplementary Table T1**. Parameters of the experimental set up and sample surface

| **Parameters** | | **Values** |
| --- | --- | --- |
| Load | | 0.5N |
| Area of the sample (contact area) | | 25 *cm^2^* |
| Frictional Surface | Roughness Average (**R_a_**) | 4.58 ± 0.25 *µm* |
|  | Root Mean Square average (**R_q_**) | 4.62 ± 0.23 *µm* |
|  | Mean Height of the Profile (**R_t_**) | - 1. 0.71 *µm* |
|  | Average Maximum  Height of the Profile (**R_z_**) | 12.96 ± 0.70 *µm* |
| Sample | Roughness (**R_z_**) | 10.18 ± 1.1 *µm* |
| Force sensor (capacity) | | 1 *kg* |
| Accelerometer (frequency) | | 201 *Hz* |
| Loading Velocity (**V_L_**) | | 2, 4, 6, 8, 10, 20, 30, 40, 60, 80, 100 *µm/s* |

**Supplementary Table T2.** Compiled datasets for Figure 12b and 12c for slow earthquakes.

| Locations | | Vp/Vs^a^ | Recurrence time (in months)^b^ | Plate Velocity (mm/yr)^c^ |
| --- | --- | --- | --- | --- |
| New Zealand (NZ) | | 2.010 | 24.098 | 20 |
| Coast Rica (CR) | | 1.943 | 21.128 | 20.2 |
| Mexico (ME) | | 1.733 | 12.015 | 35 |
| Japan South Ryukyu (SR) | | 1.650 | 7.173 | 32 |
| West Shikoku (WS) | | 1.632 | 6.203 | 65 |
| Eastern Kii Peninsula (EK) | | 1.591 | 6.068 | 62 |
| Cascadia | Wrangellia (WR) | 1.831 | 14.115 | 24 |
|  | Klamath (KL) | 1.753 | 10.035 | 42 |
|  | Siletzia (SI) | 1.891 | 21.128 | 22.5 |
|  | dip 1 | 1.800 | 14.041 | - |
|  | dip 2 | 1.759 | 6.983 | - |
|  | dip 3 | 1.730 | 4.163 | - |
|  | dip 4 | 1.691 | 2.250 | - |

^a,b^taken from Audet & Burgmann^91^; Schwartz and Rokosky^92^; Brudzinski and Allen^93^; Beroza and Ide^94^; Obara^95^; Lowry^96^; Heki and Kataoka^97^; Wallace and Beavan^98^; Jiang et al^99^.

^c^taken from Sella et al.^100^; Bird^101^; DeMets et al.^102^; McCaffrey et al.^103^; Wallace et al.^104-106^

**Supplementary Table T3.** Compiled datasets for Fig. 12d of Megathrust Earthquakes from 32 trench segments.

| Sr. no. | Trench^a^ | Mean Recurrence Time (years)^a^ | Mean plate motion (mm/year)^b^ |
| --- | --- | --- | --- |
| 1. | Alaska | 1509 | 60.5 |
| 2. | Andaman | 5415 | 30 |
| 3. | Antilles | 4223.5 | 19 |
| 4. | C.America | 1683.5 | 72.5 |
| 5. | C.Chile | 1077 | 69 |
| 6. | Cascadia | 1737.5 | 35 |
| 7. | E.Aleutian | 957.5 | 70 |
| 8. | Ecu-Colom | 1349.5 | 57.5 |
| 9. | Hikurangi | 2625 | 35 |
| 10. | Izu | 3179 | 40 |
| 11. | Japan | 536.5 | 71.5 |
| 12. | Java | 2120 | 67.5 |
| 13. | Kamchatka | 688 | 76.5 |
| 14. | Kermadec | 2785.5 | 58 |
| 15. | Kuriles | 966 | 75.5 |
| 16. | Marianas | 4077.5 | 50.5 |
| 17. | Mexico | 1622 | 51.5 |
| 18. | N.Chile | 1324.5 | 68.5 |
| 19. | Nankai | 1067.5 | 67.5 |
| 20. | New-Britain | 498 | 116 |
| 21. | New-Guinea | 540 | 112 |
| 22. | New-Hebrides | 962.5 | 78 |
| 23. | Peru | 1413 | 64 |
| 24. | Philippine | 947 | 104 |
| 25. | Ryukyu | 1072 | 83 |
| 26. | S.Chile | 4017.5 | 17 |
| 27. | Sandwich | 1076 | 72.5 |
| 28. | Solomon | 1074 | 95.5 |
| 29. | Sumatra | 1893.5 | 67.5 |
| 30. | Timor | 1286.5 | 76.5 |
| 31. | Tonga | 900 | 188.5 |
| 32. | W.Aleutian | 1032 | 74.5 |

^a^taken from McCaffrey^107^

^b^taken from Sella et al.^100^; Bird^101^; DeMets et al.^102^; McCaffrey et al.^103^; Wallace et al.^104-106^

**Supplementary Table T4.** Terminologies and abbreviations used in the study

| Terminologies | Abbreviations/symbols |
| --- | --- |
| Rate and State Friction | RSF |
| Rate, State and Temperature Friction | RSTF |
| Episodic Tremor and Slow slip | ETS |
| Slow slip and Tremor | SST |
| Very Low Frequency earthquakes | VLF |
| Force drop | $\Delta F$ |
| Recurrence time | T_r_ |
| Loading velocity | V_L_ |
| Slip Velocity | V |
| Shear force | Τ |
| Critical Slip Distance | L |
| State variable | θ |
| Spring constant | K |
| Critical Spring constant | K_C_ |
| Mean roughness | Rz |
| continuous Global Positioning System | cGPS |

**Supporting references**

1. Ranjith, K. & Rice, J. R. Stability of quasi-static slip in a single degree of freedom elastic system with rate and state dependent friction. J. Mech. Phys. Solids 47, 1207-1218 (1999).
2. Im, K., Saffer, D., Marone, C. & Avouac, J. P. Slip-rate-dependent friction as a universal mechanism for slow slip events. Nat. Geosci. 13, 705-710 (2020).
3. Kato, N. Effect of frictional heating on pre-seismic sliding: a numerical simulation using a rate-, state- and temperature-dependent friction law. Geophys. J. Int. 147, 183–188 (2001).
4. de Lorenzo, S. & Loddo, M. Effect of frictional heating and thermal advection on pre-seismic sliding: a numerical simulation using a rate-, state-and temperature-dependent friction law. J. Geodyn. 49, 1-13 (2010).
5. Beeler, N. M., Tullis, T. E. & Goldsby, D. L. Constitutive relationships and physical basis of fault strength due to flash heating. J. Geophys. Res. Solid Earth 113, B1 (2008).
6. Singh, A. K. & Singh, T. N. Stability of the rate, state and temperature-dependent friction model and its applications. Geophys. Suppl. Mon. Not. R. Astron. Soc. 205, 636–647 (2016).
7. Wang, L. & Barbot, S. Excitation of San Andreas tremors by thermal instabilities below the seismogenic zone. Science Adv. 6, eabb2057 (2020).
8. Tichelaar, B. W. & Ruff, L. J. Depth of seismic coupling along subduction zones. J. Geophys. Res. Solid Earth 98, B2, 2017-2037 (1993).
9. Segall, P. & Rice, J. R. Does shear heating of pore fluid contribute to earthquake nucleation? J. Geophys. Res. Solid Earth 111, B9 (2006).
10. Schmitt, S. V., Segall, P. & Matsuzawa, T. Shear heating‐induced thermal pressurization during earthquake nucleation. J. Geophys. Res. Solid Earth 116, B6 (2011).
11. Chester, F. M. & Higgs, N. G. Multimechanism friction constitutive model for ultrafine quartz gouge at hypocentral conditions. J. Geophys. Res. 97, B2, 1859–1870 (1992).
12. Chester, F. M. Effects of temperature on friction: Constitutive equations and experiments with quartz gouge. J. Geophys. Res. 99, B4, 7247–7261 (1994).
13. Audet, P., & Bürgmann, R. (2014). Possible control of subduction zone slow-earthquake periodicity by silica enrichment. Nature, 510(7505), 389-392.
14. Schwartz, S. Y. & Rokosky, J. M. Slow slip events and seismic tremor at circum-pacific subduction zones. *Rev. Geophys.* **45**, RG3004 (2007).
15. Brudzinski, M. R. & Allen, R. M. Segmentation in episodic tremor and slip all along Cascadia. Geology 35, 907–910 (2007).
16. Beroza, G. C. & Ide, S. Slow earthquakes and non-volcanic tremor. Annu. Rev. Earth Planet. Sci. 39, 271–296 (2011).
17. Obara, K. Characteristics and interactions between non-volcanic tremor and related slow earthquakes in the Nankai subduction zone, southwest Japan. J. Geodyn. 52, 229–248 (2011).
18. Lowry, A. R. Resonant slow fault slip in subduction zones forced by climatic load stress. Nature 442, 802–805 (2006).
19. Heki, K. & Kataoka, T. On the biannually repeating slow‐slip events at the Ryukyu Trench, southwestern Japan. J. Geophys. Res. Solid Earth 113, B11 (2008).
20. Wallace, L. M. & Beavan, J. Diverse slow slip behavior at the Hikurangi subduction margin, New Zealand. J. Geophys. Res. Solid Earth 115, B12 (2010).
21. Jiang, Y., Wdowinski, S., Dixon, T. H., Hackl, M., Protti, M. & Gonzalez, V. Slow slip events in Costa Rica detected by continuous GPS observations, 2002–2011. Geochem. Geophys. Geosyst. 13, 4 (2012).
22. Sella, G. F., Dixon, T. H. & Mao, A. REVEL: A model for recent plate velocities from space geodesy. J. Geophys. Res. 107, (2002).
23. Bird, P. An updated digital model of plate boundaries. Geochem. Geophys. Geosyst. 4, 1027 (2003).
24. DeMets, C., Gordon, R. G., Argus, D. F. & Stein, S. Effects of recent revisions to the geomagnetic reversal time scale on estimates of current plate motions. Geophys. Res. Lett. 21, 2191–2194 (1994).
25. McCaffrey, R., Wallace, L. M., Beavan, J. & Douglas, A. Slow slip events, temperature, and interseismic coupling at the Hikurangi subduction zone, New Zealand. AGU Spring Meeting Abstracts 2007, G31A-05 (2007).
26. Wallace, L. M. et al. GPS Constraints on Active Tectonics and Arc-Continent Collision in Papua New Guinea: evidence for edge-driven microplate rotations. J. Geophys. Res. 109, (2004).
27. Wallace, L. M., Beavan, J., McCaffrey, R. & Darby, D. Subduction zone coupling and tectonic block rotations in the North Island, New Zealand. J. Geophys. Res. 109, B12406 (2004).
28. Wallace, L. M., McCaffrey, R., Beavan, J. & Ellis, S. Rapid microplate rotations and back-arc rifting at the transition between collision and subduction. Geology 33, 857–860 (2005).
29. McCaffrey, R. Global frequency of magnitude 9 earthquakes. Geology 36, 263-266 (2008).
